# Wearable Artificial Intelligence for Detecting Anxiety: Systematic Review and Meta-Analysis

**DOI:** 10.2196/48754

**Published:** 2023-11-08

**Authors:** Alaa Abd-alrazaq, Rawan AlSaad, Manale Harfouche, Sarah Aziz, Arfan Ahmed, Rafat Damseh, Javaid Sheikh

**Affiliations:** 1 AI Center for Precision Health Weill Cornell Medicine-Qatar Cornell University, Qatar Foundation - Education City Doha Qatar; 2 Infectious Disease Epidemiology Group Weill Cornell Medicine-Qatar, Cornell University Qatar Foundation - Education City Doha Qatar; 3 World Health Organization Collaborating Centre for Disease Epidemiology Analytics on HIV/AIDS, Sexually Transmitted Infections, and Viral Hepatitis Weill Cornell Medicine–Qatar, Cornell University Qatar Foundation - Education City Doha Qatar; 4 Department of Computer Science and Software Engineering United Arab Emirates University Al Ain Abu Dhabi United Arab Emirates

**Keywords:** anxiety, artificial intelligence, wearable devices, machine learning, systematic review, mobile phone

## Abstract

**Background:**

Anxiety disorders rank among the most prevalent mental disorders worldwide. Anxiety symptoms are typically evaluated using self-assessment surveys or interview-based assessment methods conducted by clinicians, which can be subjective, time-consuming, and challenging to repeat. Therefore, there is an increasing demand for using technologies capable of providing objective and early detection of anxiety. Wearable artificial intelligence (AI), the combination of AI technology and wearable devices, has been widely used to detect and predict anxiety disorders automatically, objectively, and more efficiently.

**Objective:**

This systematic review and meta-analysis aims to assess the performance of wearable AI in detecting and predicting anxiety.

**Methods:**

Relevant studies were retrieved by searching 8 electronic databases and backward and forward reference list checking. In total, 2 reviewers independently carried out study selection, data extraction, and risk-of-bias assessment. The included studies were assessed for risk of bias using a modified version of the Quality Assessment of Diagnostic Accuracy Studies–Revised. Evidence was synthesized using a narrative (ie, text and tables) and statistical (ie, meta-analysis) approach as appropriate.

**Results:**

Of the 918 records identified, 21 (2.3%) were included in this review. A meta-analysis of results from 81% (17/21) of the studies revealed a pooled mean accuracy of 0.82 (95% CI 0.71-0.89). Meta-analyses of results from 48% (10/21) of the studies showed a pooled mean sensitivity of 0.79 (95% CI 0.57-0.91) and a pooled mean specificity of 0.92 (95% CI 0.68-0.98). Subgroup analyses demonstrated that the performance of wearable AI was not moderated by algorithms, aims of AI, wearable devices used, status of wearable devices, data types, data sources, reference standards, and validation methods.

**Conclusions:**

Although wearable AI has the potential to detect anxiety, it is not yet advanced enough for clinical use. Until further evidence shows an ideal performance of wearable AI, it should be used along with other clinical assessments. Wearable device companies need to develop devices that can promptly detect anxiety and identify specific time points during the day when anxiety levels are high. Further research is needed to differentiate types of anxiety, compare the performance of different wearable devices, and investigate the impact of the combination of wearable device data and neuroimaging data on the performance of wearable AI.

**Trial Registration:**

PROSPERO CRD42023387560; https://www.crd.york.ac.uk/prospero/display_record.php?RecordID=387560

## Introduction

### Background

Anxiety is defined as an unpleasant emotional state whose cause is either not easily defined or considered to be uncontrollable or unavoidable, resulting in tension and physiological manifestations [1]. Anxiety disorders (ADs) include generalized AD, social AD, panic disorder, and various phobia-related disorders [2-5]. ADs are one of the most common mental disorders, and they have a high prevalence worldwide. It is estimated that 284 million people worldwide have been diagnosed with AD [6]. A report conducted by the National Health Interview Survey revealed that 15.6% of adults in the United States had ADs in 2019 [7]. In Europe, anxiety is the most prevalent mental health condition among people aged 14 to 65 years, with a 12-month prevalence of 14% and approximately 61.5 million affected individuals [8]. Studies have also reported that AD affects 14.5% to 33.7% of the population at least once in their lifetime, which means that up to one-third of individuals experience AD at some point in their lives [9]. People with ADs often experience intense, excessive, and persistent worry and fear about everyday situations. Anxiety can significantly affect an individual’s social, occupational, and personal functioning and can interfere with daily activities such as job performance, schoolwork, and social relationships.

The diagnosis of ADs is a very complicated and challenging task. Currently, ADs are diagnosed and screened primarily through clinical observations of patients’ mental states, clinical histories, and self-report questionnaires (eg, the State-Trait Anxiety Inventory) for anxiety [10]. However, these approaches have been hampered by a number of significant limitations, such as the subjectivity and reproducibility of these methods, shortage of mental health professionals worldwide, the long time required to conduct comprehensive clinical interviews, and the extensive presence of comorbidities in patients with anxiety [11]. As a result, anxiety is commonly underdetected and undertreated despite the huge disease burden. Thus, there is a substantial need for more efficient automated tools and technologies that can overcome the challenges of the current approaches to anxiety assessment [12].

Advances in digital technologies and wireless sensors have led to the proliferation of wearable health care devices, which can be particularly useful for the diagnosis and prediction of anxiety. Wearable devices offer a convenient way for people with anxiety to monitor, examine, track, and share their health features, such as physical activities, heart rates, sleep patterns, blood oxygen, and respiratory rate. Wearable devices are made in different forms to meet their use requirements and can be classified into 4 types: on-body devices (fixed directly on the body or skin), near-body devices (fixed close to the body with no direct contact with the body or skin), in-body devices (implantable electronics), and electronic textiles (textiles with integrated electronics).

Wearable devices have undergone a significant transformation over the last few years, reflecting the rapid advancement of technology in the field. Early iterations of smartwatches and activity trackers were primarily focused on basic monitoring and display functions. Many of these devices lacked connectivity options, limiting their ability to interact with other technologies. However, the introduction of Bluetooth components marked a turning point in the evolution of wearables, allowing for synchronization with smartphones and other wireless devices. This integration not only enhanced the user experience but also paved the way for more advanced functionalities.

More recent versions of wearable devices have embraced cutting-edge innovations by incorporating artificial intelligence (AI) and machine learning components, thus introducing what we call *wearable AI* technology. Wearable AI is the fusion of data obtained from wearables and sophisticated machine learning algorithms [13]. Machine learning techniques can be used for analyzing a patient’s wearable data to detect anxiety, helping replicate human reasoning or make logical decisions. Moreover, many wearable devices come equipped with embedded computing capacity that enables them to use AI algorithms. However, other wearable devices can use another connected device or the cloud for the required computing power. Hence, resource-intensive AI algorithms can be seamlessly integrated into a wearable device [14-16]. If effectively used, wearable AI can greatly help in the accurate diagnosis and prediction of anxiety as well as the management of several ADs.

### Research Problem and Aim

In the past few years, numerous studies have examined the performance of wearable AI devices for the detection of anxiety. In an effort to summarize these studies, several reviews have been conducted, but they had the following limitations. First, most extant reviews have largely focused on general wearable devices rather than wearable AI devices [12,17-21]. Second, in many of these reviews, specific age groups were targeted, such as children and adolescents [20]. Third, a large number of these reviews did not search relevant databases such as PsycINFO [17,19,20], ACM Digital Library [17-21], and IEEE Xplore [17-21]. Fourth, some of these reviews examined the performance of wearable AI for limited data types (eg, electrocardiogram [ECG] data) [12] rather than considering all data types collected by wearables. Finally, and most importantly, no systematic reviews or meta-analyses have been conducted to evaluate the effectiveness of wearable AI in detecting anxiety [17,19,20,22]. To address this gap, this review aimed to examine the performance of wearable AI in detecting and predicting anxiety. It is worth noting that this review is built upon and differs from our previous reviews [22,23]. Specifically, the first study [22] was a scoping review to explore the features of wearable AI used for anxiety and depression and identify the research gaps in this area. However, this scoping review did not focus on the performance of wearable AI in detecting and predicting depression or anxiety [22]. The second study was a systematic review and meta-analysis that summarized the evidence on the performance of wearable AI in detecting and predicting depression [23]. This review will bridge one of the gaps identified by the first review and not addressed by the second review, which is the assessment of the performance of wearable AI in detecting and predicting anxiety.

## Methods

### Overview

The authors conducted and reported this systematic review in accordance with the PRISMA-DTA (Preferred Reporting Items for Systematic Reviews and Meta-Analyses extension for Diagnostic Test Accuracy) [24]. The PRISMA-DTA checklist for this review is outlined in Multimedia Appendix 1 [24]. The protocol for this review was registered in PROSPERO (ID: CRD42023387560).

### Search Strategy

To find relevant studies, the first author searched the following 8 electronic databases on October 3, 2022: MEDLINE (via Ovid), Embase (via Ovid), PsycINFO (via Ovid), CINAHL (via EBSCO), ACM Digital Library, Scopus, IEEE Xplore, and Google Scholar. An automated search was set up with biweekly alerts for 3 months (ending on January 2, 2023). Owing to the large number of results retrieved from Google Scholar, only the first 100 hits (ie, 10 pages) were checked for this review. To identify additional studies, we screened the reference lists of the included studies (ie, backward reference list checking) and reviewed studies that cited the included studies (ie, forward reference list checking).

The search terms used in this review were compiled after consulting with 3 experts in digital mental health and after reviewing relevant reviews. The search query was composed of 3 groups of terms: those related to AI (eg, *artificial intelligence*, *machine learning*, and *deep learning*), those related to wearable devices (eg, *wearable*, *smart watch*, and *smartwatch*), and those related to anxiety (eg, *anxiety* and *anxious*). The search queries used in this review are presented in Multimedia Appendix 2.

### Study Eligibility Criteria

This review examined papers that focused on building or applying AI algorithms for detecting or predicting anxiety using data from wearable devices. The selection criteria for articles that qualified for inclusion and exclusion were agreed upon through the collaborative expertise of the authors. To be considered for inclusion in this review, studies had to evaluate the performance of AI algorithms in detecting or predicting anxiety and report the confusion matrix or performance measures (eg, accuracy, sensitivity, or specificity). We excluded studies that used AI to predict the outcome of an anxiety intervention or treatment. The data acquisition had to be via noninvasive on-body wearables, such as smartwatches, smart glasses, smart wristbands, smart clothes, and smart rings. We excluded studies that used the following devices to collect the data: nonwearable devices, handheld devices (eg, mobile phones), near-body wearable devices (eg, devices that do not have direct contact with the body surface), in-body wearable devices (eg, implants), wearable devices wired to nonwearable devices, and wearable devices requiring expert supervision (eg, wearable devices that require placement of electrodes at very specific body points). This review included studies that collected data using other methods (eg, nonwearable devices, interviews, and questionnaires) along with wearable devices. We included peer-reviewed journal articles, conference papers, and dissertations with full text regardless of study settings, reference standards, and the country in which the study was conducted. Considering our focus on modern technology and the fact that the domain of wearable AI devices is under constant development, only articles from 2015 onward were included. Studies published in a language other than English or structured as review articles, editorials, conference abstracts, preprints, posters, protocols, and research highlights were excluded. Articles demonstrating a theoretical foundation for wearable AI devices for anxiety were disregarded.

### Study Selection

Relevant studies were identified through the following 3 steps. First, all the retrieved studies were imported into EndNote X9 (Clarivate Analytics) to identify and eliminate duplicate items. Second, 2 reviewers independently screened the titles and abstracts of all the retrieved studies. Finally, the remaining articles were subsequently sourced in full text and inspected by the 2 reviewers independently. Any disagreements in the second and third steps were resolved through discussion. The Cohen κ was used to calculate interrater agreement, which was 0.90 for title and abstract screening and 0.94 for full-text reading.

### Data Extraction

Using Excel (Microsoft Corp), 2 reviewers independently extracted metadata, wearable devices, AI algorithms, and results of the studies. The data extraction form used in this review was pilot-tested with 5 studies (Multimedia Appendix 3). Any disputes in the extracted data between the reviewers were resolved through consensus. For all studies in which raw data or confusion matrices were reported, we calculated the following performance metrics: accuracy, specificity, and sensitivity. If the confusion matrix was not available in the published studies, the first and corresponding authors were contacted in an attempt to retrieve it. We did not include results derived from AI algorithms based solely on nonwearable device data (eg, data collected by smartphones or questionnaires). As many studies conducted multiple experiments to test, for example, different numbers of features, data types, validation approaches, and AI algorithms, they reported several results for the same performance measure. Thus, for these studies, we extracted the highest results for each performance measure for each algorithm.

### Risk-of-Bias and Applicability Appraisal

To carefully assess the quality of the included studies, we adapted a well-known tool (Quality Assessment of Diagnostic Accuracy Studies–Revised; QUADAS-2) [25] for our analysis by replacing some irrelevant criteria with more relevant criteria from another applicable tool (the Prediction Model Risk of Bias Assessment Tool) [26]. In this section, we describe our modified QUADAS-2 tool that is based on both experience using the original tool and potential sources of bias originating from differences in the design and conduct of the included studies. Our QUADAS-2 modified tool consists of 4 domains: participants, index test (AI algorithms), reference standard (ground truth), and analysis. Each domain comprises 4 signaling questions that were developed to address the specific aims of this review. In addition to assessing the risk of bias for each of the 4 domains, the first 3 domains are also assessed in terms of concerns regarding applicability. In total, 2 reviewers independently examined the risk of bias in the included studies using the modified version of the QUADAS-2 (Multimedia Appendix 4), which was first trialed with 5 studies. Any inconsistencies in decisions between the reviewers were resolved through discussion.

### Data Synthesis

Narrative and statistical approaches were used to synthesize the data extracted from the included studies. In our narrative synthesis, we used text and tables to summarize and describe the characteristics of the included studies (study metadata, wearable devices, and AI techniques). With regard to the statistical approach, DerSimonian-Laird random-effects models [27] using the Freeman-Tukey double arcsine transformation [28,29] were conducted to pool outcome measures (ie, accuracy, sensitivity, and specificity) when the extracted effect sizes in one stratum were independent (ie, extracted from different unique citations). This methodology accounts for the sampling variation and heterogeneity in effect sizes and was conducted using the *meta* package in R (version 4.2.2; R Foundation for Statistical Computing) [30].

In this review, some studies reported multiple effect sizes. Such studies will have a larger effect on the results of the meta-analysis than studies reporting only one effect size. Therefore, we used a multilevel meta-analysis [27,31] to account for this dependency in effect sizes (ie, extracted from the same citation), thereby reducing the likelihood of type-I errors. Multilevel meta-analyses were conducted using the *metafor* package in R (version 4.2.2) [32].

When applicable, subgroup multilevel meta-analyses were conducted to assess for a possible association between outcome measures and different moderators (algorithms, aims of AI, wearable devices used, status of wearable devices, data types, data sources, reference standards, and validation methods [27,31]). The strength of evidence for an association was deemed significant for moderators with a *P* value of <.05.

Between-study heterogeneity was assessed using the Cochran *Q* statistic (*P*<.05 indicated heterogeneity), between-study variance was assessed using τ^2^, and the magnitude of between-study variation because of true difference in effect sizes rather than chance was assessed using *I*^2^ [29,33]. The degree of heterogeneity was considered insignificant when *I*^2^ ranged from 0% to 40%, moderate when it ranged from 30% to 60%, substantial when it ranged from 50% to 90%, or considerable when it ranged from 75% to 100% [34].

## Results

### Search Results

**Figure 1:**
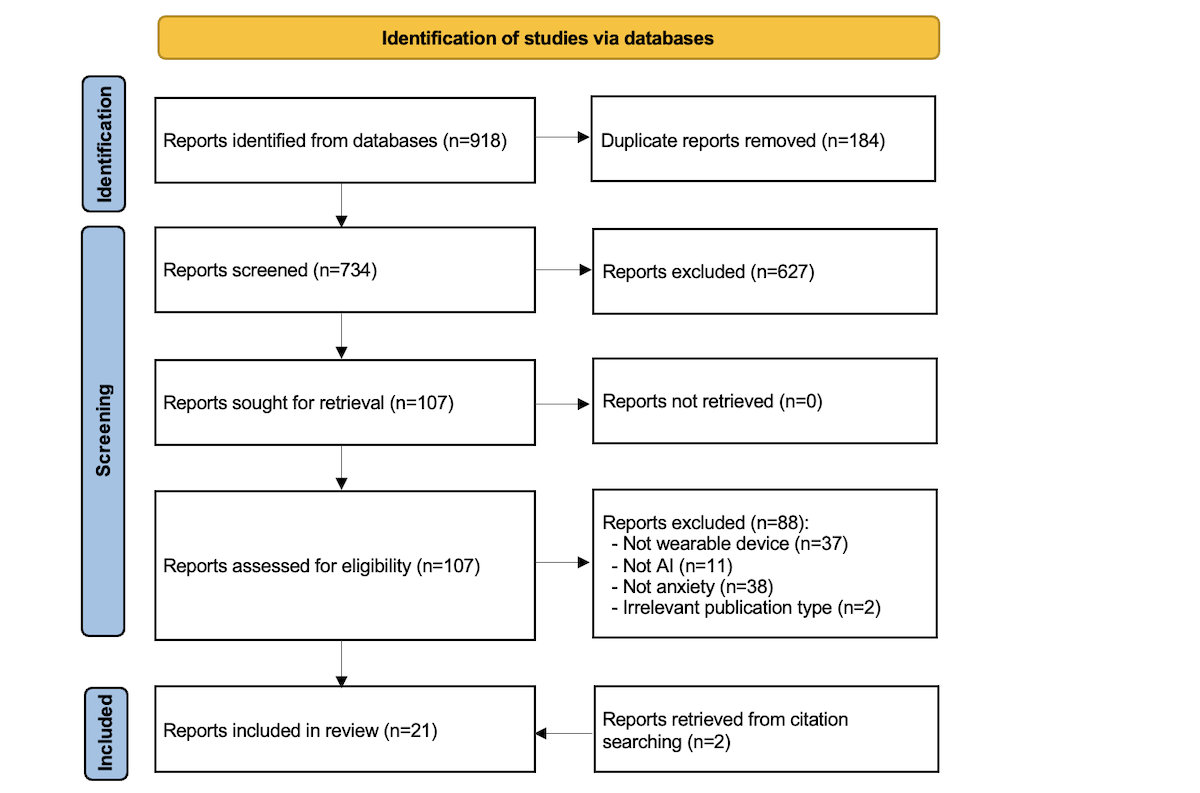
Flowchart of the study selection process. AI: artificial intelligence.

The results of the systematic search are presented in Figure 1. A total of 918 studies were identified through the systematic search across the preidentified databases. Of the 918 identified studies, 184 (20%) duplicates were removed using EndNote X9, leaving 734 (80%) studies. A further 85.4% (627/734) of the studies were excluded following title and abstract screening. We retrieved and read the full text of the remaining 107 studies. The full-text reading led to the removal of 82.2% (88/107) of the studies, primarily because of not using wearable devices, not using AI methods, not having anxiety as a measured outcome, or being other irrelevant publication types. We identified 2 additional studies relevant to this review through backward and forward reference list checking. The remaining 21 studies were included in this review [35-55], of which 17 (81%) were included in the meta-analysis [35-43,45,46,48,49,51,52,54,55].

### Characteristics of the Included Studies

The key characteristics of the studies included in the review are presented in Table 1. The included studies were published between 2016 and 2022. The years in which the largest number of included studies was published were 2021 (6/21, 29%) and 2020 (6/21, 29%). Studies were conducted in 10 different countries (Table 1), with the United States accounting for more than a quarter of the included studies (6/21, 29%). Most of the studies (15/21, 71%) were peer-reviewed journal articles, and the rest were conference papers (6/21, 29%). The number of participants in the included studies ranged from 10 to 823, with an average of 173.4 (SD 247; Table 1). The mean age of the participants was reported in more than half (11/21, 52%) of the studies and ranged from 19.8 to 73.4 years, with an average of 35.0 (SD 14.4) years. All studies targeted adults, with 5% (1/21) of the studies focusing only on older adults (aged 60-80 years). A total of 71% (15/21) of the studies reported the proportion of female participants, which ranged from 37% to 66.3%, with an average of 57.7% (SD 13.3%). Most studies (17/21, 81%) recruited participants with any health condition, and the remaining studies either focused on patients with a specific AD (4/21, 19%) or recruited both patients with anxiety and healthy individuals (1/21, 5%). The characteristics of each included study are listed in Multimedia Appendix 5 [35-55].

**Table 1:** Characteristics of the included studies (N=21).

Feature			Values		References
**Year of publication, n (%)**					
	2022	3 (14)		[42,48,54]	
	2021	6 (29)		[35,43,45,47,50,52]	
	2020	6 (29)		[36-39,41,44]	
	2019	3 (14)		[49,51,53]	
	2017	1 (5)		[40]	
	2016	2 (10)		[46,55]	
**Country of publication, n (%)**					
	United States	6 (29)		[39,42,43,47,50,53]	
	United Kingdom	3 (14)		[37,49,52]	
	Pakistan	3 (14)		[35,36,45]	
	Japan	2 (10)		[38,48]	
	China	2 (10)		[40,44]	
	Other (Germany, Hong Kong, Lithuania, Mexico, and Taiwan)	1 (5) each		[41,46,51,54,55]	
**Type of publication, n (%)**					
	Journal article	15 (71)		[35,37,40-47,50-52,54,55]	
	Conference paper	6 (29)		[36,38,39,48,49,53]	
Number of participants, mean (SD; range)			173.4 (247; 10-823)		[35-55]
Age of participants (years), mean (SD; range)			35.0 (14.4; 19.8-73.4)		[35,37,42,43,46-48,50-53]
Gender (% of women), mean (SD; range)			57.7 (13.3; 37-66.3)		[35-37,39,42-48,51-54]
**Health conditions^a^, n (%)**					
	Any health condition	17 (81)		[35-40,42-50,53,55]	
	Social anxiety	1 (5)		[52]	
	Panic disorders	1 (5)		[54]	
	Arachnophobia	1 (5)		[41]	
	Glossophobia	1 (5)		[51]	
	Healthy	1 (5)		[51]	

^a^Numbers do not add up as participants in one study had more than 1 health condition.

### Features of Wearable AI

Among the included studies, 8 different wearable devices were used. Approximately a quarter of all studies (5/21, 24%) did not indicate what type of wearable device they used. The most common wearable devices used were the Fitbit series (eg, Fitbit Charge, Fitbit Flex, and Fitbit Alta; 4/21, 19%), the Empatica series (3/21, 14%), and Muse (3/21, 14%; Table 2). There were 9 locations on the body where wearable devices were worn in the included studies; however, wrist-worn devices were the most prevalent (15/21, 71%). The included studies used AI to detect the current anxiety status in 86% (18/21) of the studies or predict the occurrence of anxiety in the future in 14% (3/21) of the studies. The AI algorithms in the included studies were used to solve classification problems (20/21, 95%), regression problems (2/21, 10%), and clustering problems (2/21, 10%). Among the included studies, 20 different algorithms were used, but the most commonly used algorithms were support vector machine (10/21, 48%) and random forest (RF; 8/21, 38%). Nearly all studies (19/21, 90%) used closed data sets (ie, collected by the authors of the study or obtained from previous studies) except for 10% (2/21) of the studies, which used open data sets (public databases). The included studies used 14 types of data to develop their models (Table 2). The most common data used to develop the models were heart rate data (eg, heart rate, heart rate variability, and interbeat interval; 12/21, 57%), physical activity data (eg, step counts, calories, and metabolic rate; 9/21, 43%), electrodermal activity data (6/21, 29%), and sleep data (eg, duration and patterns; 5/21, 24%). There were 13 different tools used by the included studies to identify the ground truth, but the State-Trait Anxiety Inventory (8/21, 38%) was the most common. Among the included studies, 3 methods were used to validate the performance of the models, which were k-fold cross-validation (13/21, 62%), hold-out cross-validation (7/21, 33%), and leave-one-out cross-validation (4/21, 19%). The features of the wearable devices in each included study are described in Multimedia Appendix 6 [35-55].

**Table 2:** Features of artificial intelligence (AI) wearables (N=21).

Feature			Studies, n (%)		References
**Wearable device^a^**					
	Fitbit series	4 (19)		[38,39,48,53]	
	Empatica series	3 (14)		[46,51,52]	
	Muse	3 (14)		[35,36,46]	
	Vivosmart	2 (10)		[50,54]	
	Other	1 (5) each		[37,41-43,46,53,55]	
	Not reported	5 (24)		[40,44,45,47,49]	
**Placement^b^**					
	Wrist	15 (71)		[37-40,43-48,50-54]	
	Head	4 (19)		[35,36,46,55]	
	Chest	2 (10)		[46,53]	
	Other (eyes, hip, neck, arm, hand, and waist)	1 (5) each		[40-42,49,55]	
**Aim of AI algorithms**					
	Detection	18 (86)		[35-38,40-42,44-48,50-53,55]	
	Prediction	3 (14)		[39,43,54]	
**Problem-solving approaches^c^**					
	Classification	20 (95)		[35-49,51-55]	
	Regression	2 (10)		[42,50]	
	Clustering	2 (10)		[39,50]	
**AI algorithms^d^**					
	Support vector machine	10 (48)		[39,41,43,46,47,49-53,55]	
	Random forest	8 (38)		[35,36,38,43,45,47,52,54]	
	Decision tree	4 (19)		[41,49,52,54]	
	K-nearest neighbor	4 (19)		[41,43,52,55]	
	Multilayer perceptron	4 (19)		[35,36,49,50]	
	Logistic regression	3 (14)		[35,36,47]	
	Long short-term memory	3 (14)		[37,44,45]	
	XGBoost	3 (14)		[43,50,54]	
	Convolutional neural network	2 (10)		[44,45]	
	Gradient boosting	2 (10)		[45,50]	
	Ensemble model	2 (10)		[41,42]	
	K-means	2 (10)		[40,50]	
	Linear discriminant analysis	2 (10)		[41,54]	
	Other	1 (5) each		[41,43,45,48,50,54]	
**Data set source**					
	Closed	19 (90)		[35-38,40-49,51-55]	
	Open	2 (10)		[39,50]	
**Data input to AI algorithm^e^**					
	Heart rate data	12 (57)		[37,39,41,46-49,51-55]	
	Physical activity data	9 (43)		[39,42-45,48-50,54]	
	Electrodermal activity data	6 (29)		[41,46,47,49,51,52]	
	Sleep data	5 (24)		[38,43,48,50,54]	
	EEG^f^ data	3 (14)		[35,36,55]	
	Audio data	2 (10)		[40,44]	
	Behavioral data	2 (10)		[48,50]	
	Skin temperature data	2 (10)		[51,52]	
	Other	1 (5) each		[48,50,54]	
**Ground truth assessment^g^**					
	STAI^h^	8 (38)		[35-37,39,40,44,47,50]	
	DAMS^i^	2 (10)		[38,48]	
	Observation	2 (10)		[41,46]	
	CIDI^j^	2 (10)		[42,43]	
	Other	1 (5) each		[37,41,44,45,51,52,54,55]	
	Not reported	2 (10)		[49,53]	
**Validation approach^k^**					
	K-fold cross-validation	13 (62)		[35,36,41-43,45,48,50-55]	
	Hold-out cross-validation	7 (33)		[37,39,44,45,47,50,54]	
	Leave-one-out cross-validation	4 (19)		[38,45,46,51]	
	Not reported	2 (10)		[40,49]	

^a^Numbers do not add up as several studies used more than 1 wearable device.

^b^Numbers do not add up as the wearable devices in 1 study were placed in different parts of the body.

^c^Numbers do not add up as many studies used more than 1 problem-solving approach.

^d^Numbers do not add up as many studies used more than 1 AI algorithm.

^e^Numbers do not add up as many studies used more than 1 data input.

^f^EEG: electroencephalogram.

^g^Numbers do not add up as many studies used more than 1 tool to assess the ground truth.

^h^STAI: State-Trait Anxiety Inventory.

^i^DAMS: Depression and Anxiety Mood Scale.

^j^CIDI: Composite International Diagnostic Interview.

^k^Numbers do not add up as many studies used more than 1 validation approach.

### Results of Risk-of-Bias Appraisal

**Figure 2:**
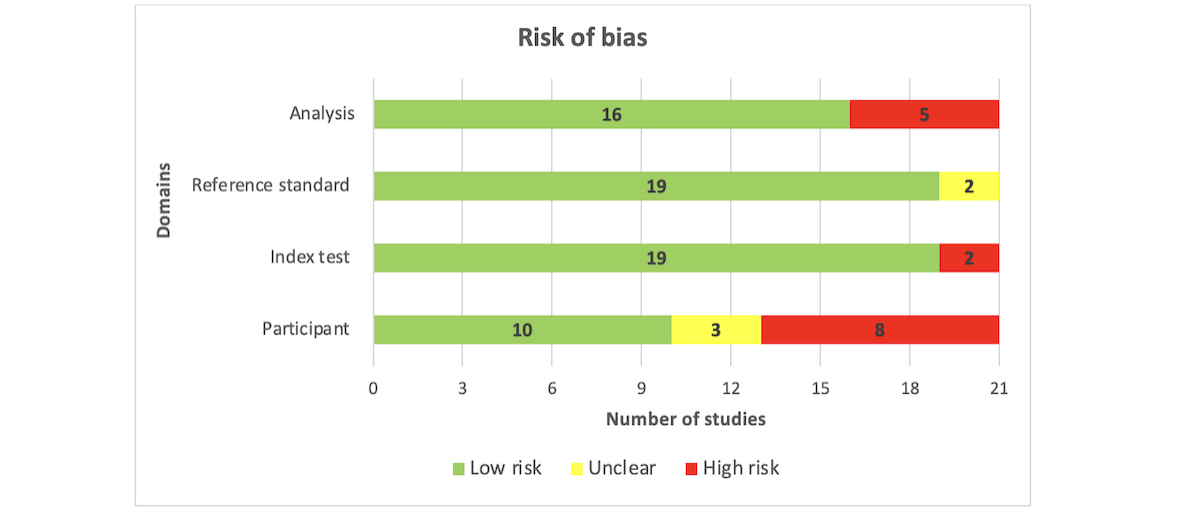
Results of the assessment of risk of bias in the included studies.

**Figure 3:**
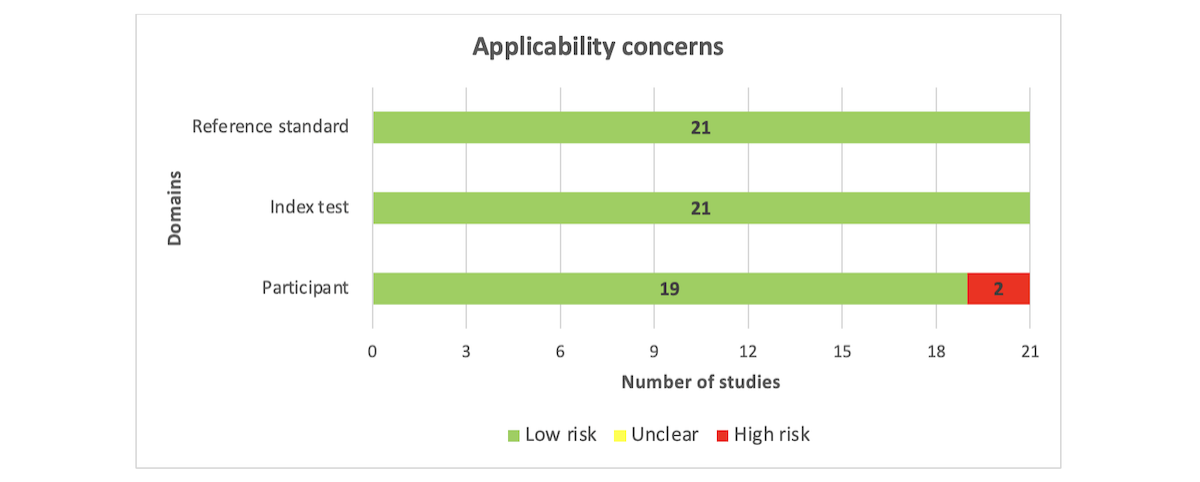
Results of the assessment of applicability concerns in the included studies.

Approximately two-thirds of the studies (14/21, 67%) did not provide adequate information to identify whether an appropriate consecutive or random sample of eligible patients was used. Most of the included studies (20/21, 95%) avoided inappropriate exclusions. The number of patients in the subgroups was appropriately balanced across half (10/21, 48%) of the studies. A sufficient sample size was reported in 43% (9/21) of the studies, whereas there was no clear indication of whether a sufficient sample size was used in the remaining studies (12/21, 57%). Consequently, the risk of bias resulting from the “selection of participants” was rated as low in only half (10/21, 48%) of the studies (Figure 2). A low level of concern was judged regarding the matching between the spectrum of participants and the prestated requirements in 90% (19/21) of the studies (Figure 3).

Almost all studies (20/21, 95%) described the AI models in detail. Most of the included studies (19/21, 90%) provided a clear description of the features (predictors) used in the models, and the features in nearly all studies (20/21, 95%) were assessed in the same way for all participants. In all the included studies (21/21, 100%), features were collected without knowledge of outcome data. Thus, the risk of bias owing to the “index test” was rated as low in most of the included studies (19/21, 90%; Figure 2). All studies (21/21, 100%) were judged to have low concerns that the definition, assessment, or timing of predictors in the model did not match the review question (Figure 3).

The outcome of interest (ie, anxiety level) was assessed using appropriate tools in 81% (17/21) of the included studies. The outcome was defined in a similar way for all participants in almost all studies (20/21, 95%) and was determined without knowledge of predictor information in all studies (21/21, 100%). An adequate interval was used between the index test and the reference standard in most studies (17/21, 81%). Accordingly, the risk of bias because of the “reference standard” was low in 90% (19/21) of the studies (Figure 2). All the included studies (21/21, 100%) were judged to have low concerns that the outcome definition, timing, or determination did not match the review question (Figure 3).

All participants enrolled in the study were included in the data analysis in 62% (13/21) of the studies. In 90% (19/21) of the studies, the data preprocessing was carried out appropriately, and in 86% (18/21) of the studies, the breakdown of the training, validation, and test sets was adequate. In 71% (15/21) of the studies, suitable measures were used to evaluate the performance of the models. According to these judgments, 76% (16/21) of the studies had a low risk of bias in the “analysis” domain (Figure 2). Multimedia Appendix 7 [35-55] shows the reviewers’ judgments on the “risk of bias” and “applicability concerns” for each domain in each included study.

### Results of the Studies

#### Overview

Meta-analyses were carried out for the highest accuracy, sensitivity, and specificity. Furthermore, when applicable, subgroup meta-analyses were performed to assess the performance of wearable AI based on different AI algorithms, aims of AI, wearable devices used, status of wearable devices, data types, data sources, reference standards, and validation methods. The following sections present the aforementioned results.

#### Accuracy

Wearable AI accuracy, which is the ability of the AI to correctly classify patients with and without anxiety, was examined in 81% (17/21) of the studies. From these investigations, we extracted 40 accuracy estimates as multiple algorithms were often assessed in a single study. The highest accuracies observed spanned 0.50 to 1.00. As displayed in Table 3, a meta-analysis of the 40 estimates derived from 149,909 participants across the 81% (17/21) of studies revealed a pooled mean accuracy of 0.82 (95% CI 0.71-0.89). The meta-analyzed evidence exhibited considerable statistical heterogeneity (*P*<.001; *I*^2^=99.9%). Table 3 also indicates that, through subgroup analyses, no statistically significant difference (*P*>.05) was found in the highest accuracy between subgroups in all groups.

**Table 3:** Pooled mean estimates of highest accuracy by several factors.

Group			Studies, N^a^		Sample size, N		Accuracy (%), range		Pooled mean accuracy, mean (95% CI)		Heterogeneity measures							Test for subgroup differences (*P* value)	
											τ^2^		Q (*P* value)		*I*^2^ (%)				
**Algorithms**																			.07
	Support vector machine	7		21,413		0.50-0.99		0.82 (0.67-0.94)		0.0520		819.0 (<.001)		99.3					
	Random forest	6		22,132		0.56-0.99		0.83 (0.68-0.94)		0.0426		1187.6 (<.001)		99.6					
	Decision tree	4		21,785		0.70-0.99		0.87 (0.68-0.98)		0.0585		1164.3 (<.001)		99.7					
	Multilayer perceptron	3		504		0.71-0.87		0.81 (0.70-0.90)		0.0087		8.3 (.02)		75.8					
	Logistic regression	2		93		0.70-0.71		0.71 (0.61-0.80)		0.0000		0.0 (.98)		0.0					
	XGBoost	2		1239		0.55-0.67		0.62 (0.50-0.73)		0.0070		12.7 (<.001)		92.1					
	Long short-term memory networks	2		10,695		0.67-0.69		0.67 (0.66-0.69)		<0.0001		1.2 (.27)		17.7					
	Ensemble model	2		605		0.91-0.94		0.92 (0.89-0.94)		0.0003		1.4 (.24)		28.6					
	K-nearest neighbor	2		61,022		0.62-0.99		0.88 (0.32-1.00)		0.1672		15.0 (<.001)		93.4					
**Aims of AI^b^**																			.33
	Detection^c^	33		143,800		0.50-0.99		0.84 (0.72-0.91)		0.2857		62,108.0 (<.001)		99.9					
	Prediction	7		6109		0.55-0.81		0.72 (0.66-0.78)		0.0082		117.9 (<.001)		94.9					
**Status of WD^d^**																			.91
	Commercial^c^	27		130,279		0.55-0.99		0.82 (0.68-0.91)		0.3345		28,205.8 (<.001)		99.9					
	Noncommercial^c^	11		16,610		0.67-0.95		0.85 (0.71-0.92)		0.0471		1363.3 (<.001)		99.4					
**WDs**																			.12
	Muse^c^	6		279		0.71-0.88		0.77 (0.67-0.85)		0.0000		9.0 (.11)		46.2					
	Empatica E4^c^	5		121,048		0.86-0.99		0.97 (0.00-0.99)		1.0715		1722.8 (<.001)		100					
	Fitbit	3		393		0.56-0.89		0.70 (0.45-0.89)		0.0453		52.4 (<.001)		96.2					
**Data sources**																			.59
	WD-based^c^	27		141,516		0.50-0.99		0.81 (0.64-0.90)		0.3498		59,871.8 (<.001)		99.9					
	WD-based and others^c^	13		8393		0.67-0.95		0.86 (0.75-0.92)		0.0552		622.7 (<.001)		98.4					
**Data types**																			.48
	Activity data^c^	8		18,619		0.67-0.94		0.88 (0.62-0.96)		0.1133		1041.3 (<.001)		99.7					
	Activity data and others^c^	12		7675		0.55-0.95		0.78 (0.57-0.90)		0.1492		573.6 (<.001)		99.1					
	EDA^e^ data and others^c^	9		122,650		0.71-0.99		0.92 (0.55-0.99)		0.6718		5870.6 (<.001)		99.9					
	EEG^f^ data^c^	6		279		0.71-0.88		0.78 (0.67-0.85)		0.0000		9.0 (.11)		46.2					
**Reference standards**																			.80
	STAI^b,g^	8		398		0.58-0.88		0.73 (0.61-0.82)		0.0087		19.5 (.006)		61.7					
	CIDI^h^	2		529		0.55-0.94		0.77 (0.33-1.00)		0.1099		117.5 (<.001)		99.1					
	DAMS^i^	2		296		0.56-0.89		0.75 (0.39-0.98)		0.0691		27.6 (<.001)		96.4					
**Validation methods**																			.41
	K-fold^c^	24		129,113		0.55-0.99		0.86 (0.70-0.94)		0.3875		24,618.6 (<.001)		99.9					
	Hold-out^c^	10		18,959		0.50-0.92		0.76 (0.57-0.87)		0.0303		901.7 (<.001)		99.3					
	Leave-one-out	2		582		0.56-0.74		0.66 (0.49-0.82)		0.0141		7.1 (.008)		86.0					
All studies^c^			40		149,909		0.50-1.00		0.82 (0.71-0.89)		0.2713		75,900.5 (<.001)		99.9		N/A^j^		

^a^Many studies were included more than once in each meta-analysis given that they assessed the performance of more than one algorithm.

^b^AI: artificial intelligence.

^c^Accuracy was pooled using the multilevel meta-analysis method.

^d^WD: wearable device.

^e^EDA: electrodermal activity.

^f^EEG: electroencephalogram.

^g^STAI: State-Trait Anxiety Inventory.

^h^CIDI: Composite International Diagnostic Interview.

^i^DAMS: Depression and Anxiety Mood Scale.

^j^N/A: not applicable.

#### Sensitivity

In 48% (10/21) of the studies, the sensitivity of wearable AI, referring to the AI’s capacity to accurately identify patients with anxiety, was examined. From these studies, we extracted 24 sensitivity estimates as many studies assessed sensitivity for more than one algorithm. The highest sensitivity in these studies ranged from 0.21 to 1.00. A meta-analysis of the 24 estimates, involving 97,794 participants from the 48% (10/21) of the studies, revealed a pooled mean sensitivity of 0.79 (95% CI 0.57-0.91), as displayed in Table 4. The statistical heterogeneity of the evidence was considerable (*P*<.001; *I*^2^=99.9%). Table 4 also demonstrates that, based on subgroup analyses, no statistically significant difference (*P*>.05) in the highest sensitivity was revealed between subgroups in all groups.

**Table 4:** Pooled mean estimates of highest sensitivity by several factors.

Group			Studies, N^a^		Sample size, N		Sensitivity (%), range		Pooled mean sensitivity, mean (95% CI^b^)		Heterogeneity measures						Test for subgroup differences (*P* value)	
											τ^2^		Q (*P* value)		*I*^2^ (%)			
**Algorithms**																		.53
	Random forest	5		10,424		0.57-0.99		0.78 (0.56-0.94)		0.0638		539.7 (<.001)		99.3				
	Support vector machine	3		37,807		0.47-1.00		0.84 (0.45-1.00)		0.1434		520.6 (<.001)		99.6				
	Decision tree	3		10,149		0.58-0.98		0.87 (0.57-1.00)		0.0884		427.7 (<.001)		99.5				
	Multilayer perceptron	3		206		0.60-0.90		0.76 (0.54-0.93)		0.0333		15.99 (<.001)		87.4				
	Logistic regression	2		47		0.63-0.71		0.66 (0.52-0.79)		0.0000		0.2 (.64)		0.0				
	XGBoost	2		359		0.21-0.85		0.52 (0.01-1.00)		0.2192		44.1 (<.001)		97.7				
**Aims of AI^c^**																		.70
	Detection^b^	17		95,770		0.47-1.00		0.82 (0.54-0.93)		0.4634		7418.4 (<.001)		99.9				
	Prediction	7		2041		0.21-0.85		0.69 (0.01-0.93)		0.2090		58.5 (<.001)		99.9				
**Status of WD^d^**																		.74
	Commercial^b^	20		97,299		0.21-1.00		0.78 (0.46-0.92)		0.4808		16,064.4 (<.001)		99.9				
	Noncommercial^b^	4		495		0.75-0.93		0.87 (0.50-0.97)		0.0864		7.3 (.06)		91.2				
**Data sources**																		.86
	WD-based^b^	15		95,313		0.47-1.00		0.80 (0.51-0.93)		0.4507		6773.9 (<.001)		100				
	WD-based and others^b^	9		2481		0.21-0.93		0.77 (0.01-0.97)		0.4715		416.5 (<.001)		99.2				
**Reference standards**																		.36
	STAI^b,e^	7		153		0.60-0.83		0.72 (0.59-0.81)		0.0000		4.4 (.62)		2.8				
	CIDI^f^	2		46		0.70-0.85		0.78 (0.63-0.91)		0.0036		1.3 (.25)		24.9				
**Validation methods**																		.34
	K-fold^b^	18		97,045		0.21-1.00		0.83 (0.48-0.95)		0.5614		14.910.3 (<.001)		100				
	Leave-one-out	2		254		0.47-0.57		0.50 (0.40-0.59)		0.0018		1.5 (.22)		33.3				
All studies^b^			24		97,794		0.21-1.00		0.79 (0.57-0.91)		0.4039		16,735.8 (<.001)		99.9		N/A^g^	

^a^Many studies were included more than once in each meta-analysis given that they assessed the performance of more than one algorithm.

^b^Sensitivity was pooled using the multilevel meta-analysis method.

^c^AI: artificial intelligence.

^d^WD: wearable device.

^e^STAI: State-Trait Anxiety Inventory.

^f^CIDI: Composite International Diagnostic Interview.

^g^N/A: not applicable.

#### Specificity

The specificity of wearable AI, which refers to the AI’s capacity to accurately identify patients without anxiety, was examined in 48% (10/21) of the studies. From these studies, we extracted 24 specificity estimates as many studies assessed specificity for more than one algorithm. The highest specificity observed spanned 0.52 to 1.00. As displayed in Table 5, a meta-analysis of the 24 estimates, derived from 45,555 participants across the 48% (10/21) of the studies, revealed a pooled mean specificity of 0.92 (95% CI 0.68-0.98). The meta-analyzed evidence exhibited considerable statistical heterogeneity (*P*<.001; *I*^2^=100%). Table 5 also indicates that, through subgroup analyses, no statistically significant difference (*P*>.05) was found in the highest specificity between subgroups in all groups.

**Table 5:** Pooled mean estimates of highest specificity by several factors.

Group			Studies, N^a^		Sample size, N		Specificity (%), range		Pooled mean specificity, mean (95% CI^b^)		Heterogeneity measures							Test for subgroup differences (*P* value)	
											τ^2^		Q (*P* value)		*I*^2^ (%)				
**Algorithms**																			.78
	Random forest	5		10,705		0.56-1.00		0.90 (0.71-1.00)		0.0658		208.5 (<.001)		98.1					
	Support vector machine	3		10,554		0.88-1.00		0.96 (0.84-1.00)		0.0325		189.6 (<.001)		98.9					
	Decision tree	3		10,895		0.77-1.00		0.95 (0.76-1.00)		0.0623		608.1 (<.001)		99.7					
	Multilayer perceptron	3		298		0.73-0.91		0.87 (0.83-0.91)		<0.0001		2.3 (.33)		11.0					
	Logistic regression	2		46		0.73-0.77		0.77 (0.63-0.88)		0.0000		0.1 (.71)		0.0					
	XGBoost	2		880		0.52-0.91		0.75 (0.31-1.00)		0.1070		150.2 (<.001)		99.3					
**Aims of AI^c^**																			.11
	Detection^b^	17		41,470		0.56-1.00		0.94 (0.65-0.99)		1.3743		42,583.8 (<.001)		100					
	Prediction^b^	7		4085		0.52-0.94		0.77 (0.01-0.97)		0.3083		361.9 (<.001)		100					
**Status of WD^d^**																			.62
	Commercial^b^	20		44,795		0.52-1.00		0.93 (0.61-0.99)		1.4583		70,885.1 (<.001)		100					
	Noncommercial^b^	4		760		0.70-0.97		0.89 (0.41-0.98)		0.0592		96.9 (<.001)		97.6					
**Data sources**																			.82
	WD-based^b^	15		40,959		0.52-1.00		0.93 (0.52-0.99)		1.5424		40,154.8 (<.001)		100					
	WD-based and others^b^	9		4596		0.77-0.97		0.90 (0.84-0.94)		0.0000		318.5 (<.001)		97.8					
**Reference standards**																			.88
	STAI^b,e^	7		148		0.70-0.91		0.83 (0.65-0.92)		0.0294		10.2 (.12)		51.9					
	CIDI^f^	2		483		0.52-0.96		0.77 (0.27-1.00)		0.1470		143.3 (<.001)		99.3					
**Validation methods**																			.50
	K-fold^b^	18		44,467		0.52-1.00		0.95 (0.53-1.00)		1.8181		68,899.9 (<.001)		100					
	Leave-one-out	2		328		0.56-0.94		0.79 (0.34-1.00)		0.1047		15.6 (<.001)		93.6					
All studies^b^			24		45,555		0.52-1.00		0.92 (0.68-0.98)		1.1844		75,736.0 (<.001)		100		N/A^g^		

^a^Many studies were included more than once in each meta-analysis given that they assessed the performance of more than one algorithm.

^b^Specificity was pooled using the multilevel meta-analysis method.

^c^AI: artificial intelligence.

^d^WD: wearable device.

^e^STAI: State-Trait Anxiety Inventory.

^f^CIDI: Composite International Diagnostic Interview.

^g^N/A: not applicable.

## Discussion

### Principal Findings

This review aimed to assess the performance of wearable AI in detecting and predicting anxiety. The results of our meta-analyses showed that wearable AI has a good but not optimal performance in detecting and predicting anxiety. To be more precise, the review revealed that wearable AI was able to correctly classify patients with and without anxiety in 81% of cases. Furthermore, we found that wearable AI has a slightly better performance in detecting individuals who do not have anxiety (92%) compared with those who do (79%). This may be attributed to the fact that the number of controls (individuals without anxiety) was larger than the number of cases (individuals with anxiety) in 78% (14/18) of the studies that reported the number of cases and controls. Therefore, the algorithms were trained on imbalanced data with more representation of control samples. This review also demonstrated that the performance of wearable AI was not moderated by algorithms, aims of AI, wearable devices used, status of wearable devices, data types, data sources, reference standards, and validation methods. This finding should be interpreted carefully given that the number of studies in most subgroup analyses was small (≥5).

As mentioned earlier, no previous reviews have examined the performance of wearable AI in detecting or predicting anxiety. However, a recent systematic review investigated the performance of wearable AI in detecting or predicting depression [23]. Although some of the findings of this review contradict those of the previous review [23], there are also some findings that are in agreement. Specifically, the specificity of wearable AI in this review (92%) and the previous review (93%) was comparable [23]. In contrast, the previous review showed higher accuracy (89% vs 81%) and sensitivity (87% vs 79%) than this review [23]. Furthermore, although the previous review demonstrated that the performance of wearable AI is moderated by the type of algorithm [23], our review showed no moderating effect of the type of algorithm on the performance of wearable AI. The aforementioned discrepancies in findings may be due to several reasons. First, although anxiety and depression are often interrelated, these disorders exhibit different signs, symptoms, and biomarkers. This differentiation extends to the detection methods applied through wearable AI. Wearable devices designed to detect anxiety might focus on indicators such as elevated heart rate, sweating, or muscle tension as these physiological responses often accompany anxiety episodes. In contrast, devices tailored for depression detection might prioritize data points such as sleep patterns, physical activity levels, or even vocal characteristics as these can provide insights into mood disorders such as depression. Although some wearable devices may have the capacity to monitor both sets of symptoms, the algorithms and interpretive models would need to be designed and calibrated differently to accurately diagnose either anxiety or depression. Second, the number of studies included in the meta-analyses was larger in the previous review than in this review (38 vs 17). Finally, although the data set size was ≥1000 in 41% (31/75) of the studies in the previous review, data set size was ≥1000 in 23% (9/40) of the studies in this review.

### Research and Practical Implications

Although this review showed that wearable AI is a promising tool for diagnosing anxiety, wearable AI is not ready to be implemented in clinical practice for the following reasons: (1) its performance in detecting patients with anxiety is not optimal at present, (2) the sample size was small (≤100) in two-thirds of the studies (14/21, 67%), and (3) only 29% (6/21) of the studies were judged to have a low risk of bias in all domains. Consequently, it is advisable to use wearable AI in conjunction with other clinical assessments and diagnostic criteria (eg, self-report surveys or clinical interviews) to detect and predict anxiety.

None of the commercial wearable devices in this review had AI embedded into them to detect anxiety. Instead, AI was embedded in a host device (eg, computers) where the data collected by wearable devices were stored. Therefore, there is a need to develop wearable devices that can promptly identify and predict anxiety, similar to those that detect stress (eg, Fitbit Charge 5, Apple Watch Series 7, and Samsung Galaxy Watch4), and are also capable of identifying specific time points during the day when anxiety levels are high, which could help users and health care providers identify causes of anxiety. We expect that this scenario could materialize in the near future, particularly with the advancements in wearable technology and the development of new chips that augment computing power.

The studies included in this review did not use neuroimaging data in addition to wearable device data to detect or predict anxiety. Neuroimaging can play an essential role in the diagnosis of anxiety by visualizing the brain and identifying structural or functional changes that may be associated with ADs [56-59]. Through techniques such as magnetic resonance imaging, positron emission tomography, and functional magnetic resonance imaging, it is possible to detect alterations in brain activity, blood flow, and connectivity that may be indicative of anxiety. For example, hyperactivity in the amygdala, an almond-shaped structure in the brain, can be associated with anxiety [57,58]. Therefore, one potential area of future research involves evaluating how effectively wearable AI technology can detect anxiety by analyzing both wearable device data and neuroimaging data.

Most studies (18/21, 86%) included in this review focused on the performance of wearable AI in identifying current anxiety status rather than forecasting the likelihood or severity of anxiety in the future. Predicting the occurrence of anxiety in the future is as important as or more important than detecting the current anxiety state as it can help develop and deliver more effective, timely, and personalized interventions. Thus, we encourage researchers to conduct additional investigations on the performance of wearable AI in predicting the occurrence of anxiety in the future.

None of the studies included in this review assessed the performance of wearable AI in distinguishing anxiety from other mental health conditions (depression, schizophrenia, and stress) or distinguishing types of anxiety (panic disorders, social AD, phobias, obsessive-compulsive disorder, and posttraumatic stress disorder). Typically, clinical practitioners rely on intricate and error-prone diagnostic methods to differentiate between various patient groups rather than merely distinguishing them from healthy individuals. As a result, additional research is necessary to examine the performance of wearable AI in distinguishing different types of anxiety and distinguishing individuals with anxiety from those with other mental disorders that exhibit comparable signs and symptoms of anxiety.

As previously stated, the sample size of two-thirds of the studies (14/21, 67%) was limited to ≤100 participants. This may have hindered the detection of potential variations in the efficacy of wearable AI technology in subgroup analyses. In addition, it may have restricted the use of certain algorithms that require a considerable amount of data to be trained and tested. We encourage researchers to undertake additional studies with larger sample sizes and extended durations to ensure adequate statistical power and enable the use of more sophisticated and efficient algorithms that require greater quantities of data.

Although the included studies used some common wearables (eg, Fitbit and Muse), they did not assess the performance of other common wearables such as Google Pixel Watch, Galaxy Watch, and Oura Ring. Furthermore, none of the included studies compared the performance of different wearable devices. Therefore, it is recommended that researchers evaluate the performance of other wearable devices and compare their efficacies.

The discrepancy between the wearable AI accuracy in detecting individuals with and without anxiety highlights the need for refining the AI algorithms to improve their performance. This could involve gathering more diverse and representative data, refining feature selection, or implementing advanced techniques to enhance the detection of anxiety among users.

There are many challenges associated with the integration of AI into wearable devices for mental disorders in general and ADs in particular. First, obtaining high-quality data is difficult with wearable technology owing to differences in spatial, temporal, and data resolution. This becomes more challenging when multiple devices have to be combined to collect multiple types of data to generate a comprehensive picture of the body. Therefore, the quality of wearable data should be emphasized to improve the performance of the algorithms. To achieve this, there is a need for more practical standards for wearable device development that are necessary to ensure the consistent measurement of different signals generated from wearable devices. Second, the presence of missing data, outliers, signal noise, and artifacts can also lead to large variations and inaccurate algorithms [60]. For example, it is necessary for sensors that monitor heart rate during physical activity to be able to distinguish artifacts caused by arm motion [61,62]. Furthermore, even when high-quality data are collected, transmission from wearables to processing platforms (eg, the cloud or another computing device) for processing is resource and time intensive. Therefore, the development of more sophisticated sensors that can accurately and efficiently collect and transmit cleaner data is required. In addition, more focus should be placed on building high-performing yet efficient AI algorithms to effectively handle missing data, outliers, and noise to enhance their practicality for implementation on edge-sensing devices. Overcoming these obstacles will enable AI-driven wearable devices to manage personalized anxiety, ultimately improving mental health outcomes for individuals.

The transition of wearable AI into existing clinical practice for anxiety detection and management is an intricate process that requires careful consideration. A robust framework must be devised that outlines how wearable AI technologies can complement traditional methods such as interviews, self-report surveys, and existing diagnostic criteria. Such an integration framework would involve validating AI algorithms using established clinical guidelines, ensuring data privacy and security compliance, training health care providers in the interpretation of AI-generated insights, and creating a clear protocol for incorporating these insights into patient care. The integration of wearable AI into existing practices could offer a more refined, real-time understanding of anxiety levels, allow for tailored interventions, and foster collaboration between health care providers and technology developers. Efforts toward these integrations could form a promising direction for future research and innovation, contributing to a more effective and patient-centric approach to anxiety management.

Recently, various studies have proposed statistical and AI approaches for wearable devices to study the effectiveness of various parameters and biosignals (eg, electroencephalography [EEG] and ECG) in differentiating patients with ADs from healthy individuals [22,63-66]. Automated systems have been proposed for the diagnosis and detection of such neuropsychological issues, providing more feasibility for integration with various wearable devices [35,64,67,68]. Al Zoubi et al [63] conducted an association study to explore the link between EEG microstate dynamic patterns and mood disorders and ADs. Abnormalities of the EEG microstates in mood disorders and ADs were described, with statistical significance, based on the occurrence sequence and temporal dynamics of EEG microstate signals. In another study [67], various machine learning schemes (eg, support vector machine and RF) were investigated for classification using the EEG signals of 23 patients recorded during exposure therapy with an EMOTIV EPOC wireless headset. The EEG channels exploited in the classifier were selected to ensure their statistical significance using *t* test and ANOVA based on their power spectral density. The highest accuracies of 94.9% and 92.74% using an RF classifier were achieved from the 2 and 4 levels in the power spectral density of the EEG recording, respectively. In a study carried out by Arsalan and Majid [35], EEG data acquisition was performed using an Interaxon Muse wearable headband consisting of 4 dry electrodes at positions TP9, AF7, AF8, and TP10. A classification accuracy of 78.5% and 78.5% was demonstrated using features from all 4 channels with the RF algorithm. Furthermore, an improved accuracy of 89.28% was achieved when a feature vector of length 3 was used. Some studies have suggested that ECG signals represent an optimal biosignal for automated detection and characterization of anxiety [68-70]. In another study [69], a consumer-friendly heart rate variability biofeedback wearable device was evaluated with a remote stress management coach to reduce the symptoms of anxiety. In a study carried out by Tripathy et al [68], a wearable sensor–based ECG signal was used to detect and classify the level of anxiety (light, moderate, and severe) based on features obtained using the Fourier-Bessel domain adaptive wavelet transform. The results demonstrated a superior performance of the XGBoost model with an accuracy and *F*_1_-score of 92.27% and 92.13%, respectively.

### Limitations

This review cannot comment on (1) the performance of wearable AI in diagnosing other mental disorders (eg, depression, stress, bipolar disorder, and schizophrenia); (2) the performance of wearable AI in managing anxiety or predicting outcomes of anxiety treatment; and (3) the performance of nonwearable devices, handheld devices, near-body wearable devices, in-body wearable devices, wearable devices connected to nonwearable devices using wires, and wearable devices that require an expert to be applied on users. This is because such disorders, outcomes, and wearable devices were beyond the scope of this review, thus limiting the generalizability of our findings to these contexts. In addition, the results of our meta-analyses are likely to be overestimated or underestimated for 2 reasons. First, it is probable that we overlooked some studies as our search was limited to research published in the English language from 2015 onward and we did not use terms related to types of anxiety (eg, phobia, obsessive-compulsive disorder, and posttraumatic stress disorder). Second, several studies in this review were not included in the meta-analyses as they did not provide findings suitable for meta-analysis.

### Conclusions

Although wearable AI shows promise in detecting and predicting anxiety, it is not yet advanced enough to be used in clinical practice. As such, wearable AI should be used along with other clinical assessments and diagnostic criteria to provide a more comprehensive understanding of a patient’s condition until further evidence shows an ideal performance of wearable AI. Wearable device companies should develop devices that can promptly detect anxiety and identify specific time points during the day when anxiety levels are high. There is a need to investigate the effect of using a combination of wearable device data and neuroimaging data on the performance of wearable AI in detecting and predicting anxiety. In addition, further studies are needed to differentiate among types of anxiety and differentiate patients with anxiety from those with other mental disorders. We urge researchers to compare the performance of different wearable devices in detecting anxiety.

## Appendix

Multimedia Appendix 1 PRISMA-DTA (Preferred Reporting Items for Systematic Reviews and Meta-Analyses extension for Diagnostic Test Accuracy) checklist.

Multimedia Appendix 2 Search strategy.

Multimedia Appendix 3 Data extraction form.

Multimedia Appendix 4 Modified version of the Quality Assessment of Diagnostic Accuracy Studies–Revised.

Multimedia Appendix 5 Characteristics of each included study.

Multimedia Appendix 6 Features of wearable artificial intelligence.

Multimedia Appendix 7 Reviewers’ judgments on each “risk of bias” and applicability domain for each included study.

## Abbreviations

AD: anxiety disorder
AI: artificial intelligence
ECG: electrocardiogram
EEG: electroencephalography
PRISMA-DTA: Preferred Reporting Items for Systematic Reviews and Meta-Analyses extension for Diagnostic Test Accuracy
QUADAS-2: Quality Assessment of Diagnostic Accuracy Studies–Revised
RF: random forest

## Data Availability

The data that support the findings of this study are available from the corresponding author upon reasonable request.

## References

[1] Semple D, Smyth R (2013). Oxford Handbook of Psychiatry. 3rd edition.

[2] Baxter AJ, Scott KM, Vos T, Whiteford HA (2013). Global prevalence of anxiety disorders: a systematic review and meta-regression. Psychol Med.

[3] COVID-19 Mental Disorders Collaborators (2021). Global prevalence and burden of depressive and anxiety disorders in 204 countries and territories in 2020 due to the COVID-19 pandemic. Lancet.

[4] Anxiety disorders. National Institute of Mental Health.

[5] Shahbazi F, Shahbazi M, Poorolajal J (2022). Association between socioeconomic inequality and the global prevalence of anxiety and depressive disorders: an ecological study. Gen Psychiatr.

[6] Dattani S, Rodés-Guirao L, Ritchie H, Roser M (2018). Mental health. Our World In Data.

[7] Zablotsky B, Weeks JD, Terlizzi EP, Madans JH, Blumberg SJ (2022). Assessing anxiety and depression: a comparison of national health interview survey measures. Natl Health Stat Report.

[8] Wittchen HU, Jacobi F, Rehm J, Gustavsson A, Svensson M, Jönsson B, Olesen J, Allgulander C, Alonso J, Faravelli C, Fratiglioni L, Jennum P, Lieb R, Maercker A, van Os J, Preisig M, Salvador-Carulla L, Simon R, Steinhausen HC (2011). The size and burden of mental disorders and other disorders of the brain in Europe 2010. Eur Neuropsychopharmacol.

[9] Bandelow B, Michaelis S (2015). Epidemiology of anxiety disorders in the 21st century. Dialogues Clin Neurosci.

[10] Bystritsky A, Khalsa SS, Cameron ME, Schiffman J (2013). Current diagnosis and treatment of anxiety disorders. P T.

[11] Phillips KA, Friedman MJ, Stein DJ, Craske M (2010). Special DSM-V issues on anxiety, obsessive-compulsive spectrum, posttraumatic, and dissociative disorders. Depress Anxiety.

[12] Elgendi M, Menon C (2019). Assessing anxiety disorders using wearable devices: challenges and future directions. Brain Sci.

[13] Nahavandi D, Alizadehsani R, Khosravi A, Acharya UR (2022). Application of artificial intelligence in wearable devices: opportunities and challenges. Comput Methods Programs Biomed.

[14] Wilmink G, Dupey K, Alkire S, Grote J, Zobel G, Fillit HM, Movva S (2020). Artificial intelligence-powered digital health platform and wearable devices improve outcomes for older adults in assisted living communities: pilot intervention study. JMIR Aging.

[15] Mäkynen M, Schlindwein FS (2022). Wearable devices combined with artificial intelligence-a future technology for atrial fibrillation detection?. Sensors (Basel).

[16] Hijazi H, Abu Talib M, Hasasneh A, Bou Nassif A, Ahmed N, Nasir Q (2021). Wearable devices, smartphones, and interpretable artificial intelligence in combating COVID-19. Sensors (Basel).

[17] Long N, Lei Y, Peng L, Xu P, Mao P (2022). A scoping review on monitoring mental health using smart wearable devices. Math Biosci Eng.

[18] Hickey BA, Chalmers T, Newton P, Lin C, Sibbritt D, McLachlan CS, Clifton-Bligh R, Morley J, Lal S (2021). Smart devices and wearable technologies to detect and monitor mental health conditions and stress: a systematic review. Sensors (Basel).

[19] Kang M, Chai K (2022). Wearable sensing systems for monitoring mental health. Sensors (Basel).

[20] Welch V, Wy TJ, Ligezka A, Hassett LC, Croarkin PE, Athreya AP, Romanowicz M (2022). Use of mobile and wearable artificial intelligence in child and adolescent psychiatry: scoping review. J Med Internet Res.

[21] Juchems P (2022). The use of wearable devices in the treatment and detection of anxiety: a systematic scoping review. University of Twente.

[22] Abd-Alrazaq A, AlSaad R, Aziz S, Ahmed A, Denecke K, Househ M, Farooq F, Sheikh J (2023). Correction: wearable artificial intelligence for anxiety and depression: scoping review. J Med Internet Res.

[23] Abd-Alrazaq A, AlSaad R, Shuweihdi F, Ahmed A, Aziz S, Sheikh J (2023). Systematic review and meta-analysis of performance of wearable artificial intelligence in detecting and predicting depression. NPJ Digit Med.

[24] McInnes MD, Moher D, Thombs BD, McGrath TA, Bossuyt PM, Clifford T, Cohen JF, Deeks JJ, Gatsonis C, Hooft L, Hunt HA, Hyde CJ, Korevaar DA, Leeflang MM, Macaskill P, Reitsma JB, Rodin R, Rutjes AW, Salameh JP, Stevens A, Takwoingi Y, Tonelli M, Weeks L, Whiting P, Willis BH, the PRISMA-DTA Group (2018). Preferred reporting items for a systematic review and meta-analysis of diagnostic test accuracy studies: the PRISMA-DTA statement. JAMA.

[25] Whiting PF, Rutjes AW, Westwood ME, Mallett S, Deeks JJ, Reitsma JB, Leeflang MM, Sterne JA, Bossuyt PM (2011). QUADAS-2: a revised tool for the quality assessment of diagnostic accuracy studies. Ann Intern Med.

[26] Wolff RF, Moons KG, Riley RD, Whiting PF, Westwood M, Collins GS, Reitsma JB, Kleijnen J, Mallett S (2019). PROBAST: a tool to assess the risk of bias and applicability of prediction model studies. Ann Intern Med.

[27] Ebert D, Harrer M, Cuijpers P, Furukawa T (2021). Doing Meta-Analysis with R: A Hands-On Guide.

[28] Freeman MF, Tukey JW (1950). Transformations related to the angular and the square root. Ann Math Statist.

[29] Borenstein MH, Hedges LV, Higgins JP, Rothstein HR (2009). Introduction to Meta‐Analysis.

[30] Schwarzer G (2007). Meta: an R package for meta-analysis. R News.

[31] Assink M, Wibbelink CJ (2016). Fitting three-level meta-analytic models in R: a step-by-step tutorial. Quant Meth Psych.

[32] Viechtbauer W (2010). Conducting meta-analyses in R with the metafor package. J Stat Softw.

[33] Higgins JP, Thompson SG, Deeks JJ, Altman DG (2003). Measuring inconsistency in meta-analyses. BMJ.

[34] Deeks JJ, Higgins JP, Altman DG, Higgins JP, Thomas J, Chandler J, Cumpston M, Li T, Page MJ, Welch VA, on behalf of the Cochrane Statistical Methods Group (2019). Analysing data and undertaking meta-analyses. Cochrane Handbook for Systematic Reviews of Interventions.

[35] Arsalan A, Majid M (2021). A study on multi-class anxiety detection using wearable EEG headband. J Ambient Intell Human Comput.

[36] Arsalan A, Majid M, Anwar SM (2020). Electroencephalography based machine learning framework for anxiety classification. Proceedings of the 1st International Conference on Intelligent Technologies and Applications.

[37] Coutts LV, Plans D, Brown AW, Collomosse J (2020). Deep learning with wearable based heart rate variability for prediction of mental and general health. J Biomed Inform.

[38] Fukuda S, Matsuda Y, Tani Y, Arakawa Y, Yasumoto K (2020). Predicting depression and anxiety mood by wrist-worn sleep sensor. Proceedings of the 2020 IEEE International Conference on Pervasive Computing and Communications Workshops.

[39] Feng T, Narayanan SS (2020). Modeling behavioral consistency in large-scale wearable recordings of human bio-behavioral signals. Proceedings of the 2020 IEEE International Conference on Acoustics, Speech and Signal Processing.

[40] Gu J, Gao B, Chen Y, Jiang L, Gao Z, Ma X, Ma Y, Woo WL, Jin J (2017). Wearable social sensing: content-based processing methodology and implementation. IEEE Sensors J.

[41] Ihmig FR, Neurohr-Parakenings F, Schäfer SK, Lass-Hennemann J, Michael T (2020). On-line anxiety level detection from biosignals: machine learning based on a randomized controlled trial with spider-fearful individuals. PLoS One.

[42] Jacobson NC, Feng B (2022). Digital phenotyping of generalized anxiety disorder: using artificial intelligence to accurately predict symptom severity using wearable sensors in daily life. Transl Psychiatry.

[43] Jacobson NC, Lekkas D, Huang R, Thomas N (2021). Deep learning paired with wearable passive sensing data predicts deterioration in anxiety disorder symptoms across 17-18 years. J Affect Disord.

[44] Jin J, Gao B, Yang S, Zhao B, Luo L, Woo WL (2020). Attention-block deep learning based features fusion in wearable social sensor for mental wellbeing evaluations. IEEE Access.

[45] Khan NS, Ghani MS, Anjum G (2021). ADAM-sense: anxiety-displaying activities recognition by motion sensors. Pervasive Mob Comput.

[46] Miranda D, Favela J, Ibarra C, Cruz N (2016). Naturalistic enactment to elicit and recognize caregiver state anxiety. J Med Syst.

[47] Nath RK, Thapliyal H (2021). Machine learning-based anxiety detection in older adults using wristband sensors and context feature. SN Comput Sci.

[48] Nishimura Y, Hossain T, Sano A, Arakawa Y, Inoue S, Ahad A, Inoue S, Roggen D, Fujinami K (2022). Toward the analysis of office workers’ mental indicators based on wearable, work activity, and weather data. Sensor- and Video-Based Activity and Behavior Computing.

[49] Rother R, Sun Y, Lo B (2019). Internet of things based pervasive sensing of psychological anxiety via wearable devices under natu ralistic settings. Proceedings of the 2019 Living in the Internet of Things.

[50] Saha K, Grover T, Mattingly SM, swain VD, Gupta P, Martinez GJ, Robles-Granda P, Mark G, Striegel A, De Choudhury M (2021). Person-centered predictions of psychological constructs with social media contextualized by multimodal sensing. Proc ACM Interact Mob Wearable Ubiquitous Technol.

[51] Šalkevicius J, Damaševičius R, Maskeliunas R, Laukienė I (2019). Anxiety level recognition for virtual reality therapy system using physiological signals. Electronics.

[52] Shaukat-Jali R, van Zalk N, Boyle DE (2021). Detecting subclinical social anxiety using physiological data from a wrist-worn wearable: small-scale feasibility study. JMIR Form Res.

[53] Tiwari A, Cassani R, Narayanan S, Falk TH (2019). A comparative study of stress and anxiety estimation in ecological settings using a smart-shirt and a smart-bracelet. Proceedings of the 41st Annual International Conference of the IEEE Engineering in Medicine and Biology Society.

[54] Tsai CH, Chen PC, Liu DS, Kuo YY, Hsieh TT, Chiang DL, Lai F, Wu CT (2022). Panic attack prediction using wearable devices and machine learning: development and cohort study. JMIR Med Inform.

[55] Zheng Y, Wong TC, Leung BH, Poon CC (2016). Unobtrusive and multimodal wearable sensing to quantify anxiety. IEEE Sensors J.

[56] Engel K, Bandelow B, Gruber O, Wedekind D (2009). Neuroimaging in anxiety disorders. J Neural Transm (Vienna).

[57] Holzschneider K, Mulert C (2011). Neuroimaging in anxiety disorders. Dialogues Clin Neurosci.

[58] Paulus MP (2008). The role of neuroimaging for the diagnosis and treatment of anxiety disorders. Depress Anxiety.

[59] Madonna D, Delvecchio G, Soares JC, Brambilla P (2019). Structural and functional neuroimaging studies in generalized anxiety disorder: a systematic review. Braz J Psychiatry.

[60] Fuller D, Colwell E, Low J, Orychock K, Tobin MA, Simango B, Buote R, Van Heerden D, Luan H, Cullen K, Slade L, Taylor NG (2020). Reliability and validity of commercially available wearable devices for measuring steps, energy expenditure, and heart rate: systematic review. JMIR Mhealth Uhealth.

[61] Xie J, Wen D, Liang L, Jia Y, Gao L, Lei J (2018). Evaluating the validity of current mainstream wearable devices in fitness tracking under various physical activities: comparative study. JMIR Mhealth Uhealth.

[62] Bent B, Goldstein BA, Kibbe WA, Dunn JP (2020). Investigating sources of inaccuracy in wearable optical heart rate sensors. NPJ Digit Med.

[63] Al Zoubi O, Mayeli A, Tsuchiyagaito A, Misaki M, Zotev V, Refai H, Paulus M, Bodurka J (2019). EEG microstates temporal dynamics differentiate individuals with mood and anxiety disorders from healthy subjects. Front Hum Neurosci.

[64] Ancillon L, Elgendi M, Menon C (2022). Machine learning for anxiety detection using biosignals: a review. Diagnostics (Basel).

[65] Israel SA, Irvine JM, Cheng A, Wiederhold MD, Wiederhold BK (2005). ECG to identify individuals. Pattern Recognit.

[66] Murphy L, Nakamura R, Gentile-Solomon J, Spake A, Szlosek D (2022). Assessment of age, gender, and anxiety on ECG waveform morphology in a large population of domestic dogs. Sci Rep.

[67] Muhammad F, Al-Ahmadi S (2022). Human state anxiety classification framework using EEG signals in response to exposure therapy. PLoS One.

[68] Tripathy RK, Dash DK, Ghosh SK, Pachori RB (2023). Detection of different stages of anxiety from single-channel wearable ECG sensor signal using Fourier–Bessel domain adaptive wavelet transform. IEEE Sens Lett.

[69] Chung AH, Gevirtz RN, Gharbo RS, Thiam MA, Ginsberg J (2021). Pilot study on reducing symptoms of anxiety with a heart rate variability biofeedback wearable and remote stress management coach. Appl Psychophysiol Biofeedback.

[70] Elgendi M, Menon C (2020). Machine learning ranks ECG as an optimal wearable biosignal for assessing driving stress. IEEE Access.

