# Supplementary material for: Wearable Artificial Intelligence for Detecting Anxiety: Systematic Review and Meta-Analysis
Source: J Med Internet Res. 2023 Nov 8;25:e48754. doi: 10.2196/48754 (PMC10666012; doi:10.2196/48754)
Supplement: Multimedia Appendix 2 [file jmir_v25i1e48754_app2.docx]

**Supplementary Table 2: Search strategy**

Database(s): **Ovid MEDLINE(R) ALL**1946 to October 03, 2022.

| **#** | **Searches** | **Results** |
| --- | --- | --- |
| 1 | exp Artificial Intelligence/ | 147056 |
| 2 | "Artificial Intelligence".tw. | 19111 |
| 3 | exp Machine Learning/ | 45132 |
| 4 | "Machine Learning".tw. | 55796 |
| 5 | exp Deep Learning/ | 11216 |
| 6 | "Deep Learning".tw. | 26682 |
| 7 | "supervised learning".tw. | 3641 |
| 8 | "unsupervised learning".tw. | 1715 |
| 9 | "reinforcement learning".tw. | 4214 |
| 10 | "Decision tree".tw. | 10101 |
| 11 | "K-Nearest Neighbor*".tw. | 3833 |
| 12 | "Support vector machine*".tw. | 20729 |
| 13 | "Recurrent neural network*".tw. | 3179 |
| 14 | "convolutional neural network*".tw. | 15429 |
| 15 | "Artificial neural network*".tw. | 14226 |
| 16 | "Deep Neural Networks".tw. | 3037 |
| 17 | "Naïve Bayes".tw. | 3 |
| 18 | "Naive Bayes".tw. | 2376 |
| 19 | "Fuzzy Logic".tw. | 2155 |
| 20 | "K-Means".tw. | 5574 |
| 21 | "Random Forest".tw. | 13210 |
| 22 | "Long Short-Term Memory Networks".tw. | 138 |
| 23 | autoencoder.tw. | 1529 |
| 24 | "boltzmann machine".tw. | 262 |
| 25 | "deep belief network".tw. | 252 |
| 26 | "Gradient Boost*".tw. | 3008 |
| 27 | AdaBoost.tw. | 963 |
| 28 | "Multilayer Perceptron".tw. | 1939 |
| 29 | "Ensemble learning".tw. | 1147 |
| 30 | exp Wearable Electronic Devices/ | 16804 |
| 31 | wearable*.tw. | 19321 |
| 32 | "smart watch*".tw. | 182 |
| 33 | smartwatch*.tw. | 669 |
| 34 | acceleromet*.tw. | 19744 |
| 35 | gyroscop*.tw. | 2138 |
| 36 | "inertial sensor".tw. | 994 |
| 37 | "inertial measurement unit*".tw. | 2192 |
| 38 | "fitness band*".tw. | 20 |
| 39 | "flexible band*".tw. | 87 |
| 40 | headband*.tw. | 310 |
| 41 | "head band*".tw. | 72 |
| 42 | wristband*.tw. | 630 |
| 43 | "smart insole*".tw. | 40 |
| 44 | "Smart armband".tw. | 1 |
| 45 | bracelet*.tw. | 599 |
| 46 | Emotiv.tw. | 80 |
| 47 | NeuroSky.tw. | 15 |
| 48 | Mindo.tw. | 44 |
| 49 | StarLab.tw. | 2 |
| 50 | EmSense.tw. | 0 |
| 51 | "B-Alert X24".tw. | 0 |
| 52 | Enobio.tw. | 6 |
| 53 | BrainBit.tw. | 0 |
| 54 | NeuroSky.tw. | 15 |
| 55 | Muse.tw. | 568 |
| 56 | OpenBCI.tw. | 14 |
| 57 | Neuroelectrics.tw. | 2 |
| 58 | "G.tec nautilus".tw. | 0 |
| 59 | BioSemi.tw. | 24 |
| 60 | "mBrainTrain".tw. | 2 |
| 61 | Cognionics.tw. | 4 |
| 62 | "CGX QUICK".tw. | 0 |
| 63 | Fitbit.tw. | 934 |
| 64 | Garmin.tw. | 213 |
| 65 | "Misfit shine".tw. | 23 |
| 66 | "Polar loop".tw. | 31 |
| 67 | Jawbone.tw. | 804 |
| 68 | Geneactiv.tw. | 155 |
| 69 | Empatica.tw. | 71 |
| 70 | Amiigo.tw. | 0 |
| 71 | Actigraph.tw. | 3259 |
| 72 | "Apple Watch".tw. | 209 |
| 73 | Withings.tw. | 58 |
| 74 | Sensewear.tw. | 525 |
| 75 | PowerWatch.tw. | 0 |
| 76 | "Samsung Galaxy Watch".tw. | 4 |
| 77 | Airofit.tw. | 3 |
| 78 | Amazfit.tw. | 5 |
| 79 | VivaLINK.tw. | 0 |
| 80 | "Wellue DuoEK".tw. | 0 |
| 81 | KardiaMobile.tw. | 31 |
| 82 | "Philips Biosensor".tw. | 1 |
| 83 | Biovitals.tw. | 4 |
| 84 | exp Mood Disorder/ | 103245 |
| 85 | mood disorder*.tw. | 230732 |
| 86 | exp Anxiety/ | 103156 |
| 87 | anxiety.tw. | 230625 |
| 88 | anxious.tw. | 18995 |
| 89 | 1 or 2 or 3 or 4 or 5 or 6 or 7 or 8 or 9 or 10 or 11 or 12 or 13 or 14 or 15 or 16 or 17 or 18 or 19 or 20 or 21 or 22 or 23 or 24 or 25 or 26 or 27 or 28 or 29 | 233142 |
| 90 | 30 or 31 or 32 or 33 or 34 or 35 or 36 or 37 or 38 or 39 or 40 or 41 or 42 or 43 or 44 or 45 or 46 or 47 or 48 or 49 or 50 or 51 or 52 or 53 or 54 or 55 or 56 or 57 or 58 or 59 or 60 or 61 or 62 or 63 or 64 or 65 or 66 or 67 or 68 or 69 or 70 or 71 or 72 or 73 or 74 or 75 or 76 or 77 or 78 or 79 or 80 or 81 or 82 or 83 | 57765 |
| 91 | 84 or 85 or 86 or 87 or 88 | 1544510 |
| 92 | 89 and 90 and 91 | 143 |
| 93 | limit 92 to (english language and yr="2015 -Current") | 135 |

Database(s): **Embase**1996 to 2022 Week 41.

| **#** | **Searches** | **Results** |
| --- | --- | --- |
| 1 | exp Artificial Intelligence/ | 60065 |
| 2 | "Artificial Intelligence".tw. | 22589 |
| 3 | exp Machine Learning/ | 307253 |
| 4 | "Machine Learning".tw. | 65455 |
| 5 | exp Deep Learning/ | 24222 |
| 6 | "Deep Learning".tw. | 30305 |
| 7 | "supervised learning".tw. | 4168 |
| 8 | "unsupervised learning".tw. | 1992 |
| 9 | "reinforcement learning".tw. | 4871 |
| 10 | "Decision tree".tw. | 14273 |
| 11 | "K-Nearest Neighbor*".tw. | 4679 |
| 12 | "Support vector machine*".tw. | 25140 |
| 13 | "Recurrent neural network*".tw. | 3588 |
| 14 | "convolutional neural network*".tw. | 18175 |
| 15 | "Artificial neural network*".tw. | 16577 |
| 16 | "Deep Neural Networks".tw. | 3396 |
| 17 | "Naïve Bayes".tw. | 17 |
| 18 | "Naive Bayes".tw. | 3021 |
| 19 | "Fuzzy Logic".tw. | 2679 |
| 20 | "K-Means".tw. | 7642 |
| 21 | "Random Forest".tw. | 16726 |
| 22 | "Long Short-Term Memory Networks".tw. | 308 |
| 23 | autoencoder.tw. | 1838 |
| 24 | "boltzmann machine".tw. | 464 |
| 25 | "deep belief network".tw. | 458 |
| 26 | "Gradient Boost*".tw. | 3835 |
| 27 | AdaBoost.tw. | 1324 |
| 28 | "Multilayer Perceptron".tw. | 2237 |
| 29 | "Ensemble learning".tw. | 1474 |
| 30 | exp Wearable Electronic Devices/ | 6513 |
| 31 | wearable*.tw. | 21167 |
| 32 | "smart watch*".tw. | 261 |
| 33 | smartwatch*.tw. | 928 |
| 34 | acceleromet*.tw. | 24388 |
| 35 | gyroscop*.tw. | 2214 |
| 36 | "inertial sensor".tw. | 1232 |
| 37 | "inertial measurement unit*".tw. | 2475 |
| 38 | "fitness band*".tw. | 24 |
| 39 | "flexible band*".tw. | 268 |
| 40 | headband*.tw. | 518 |
| 41 | "head band*".tw. | 90 |
| 42 | wristband*.tw. | 1024 |
| 43 | "smart insole*".tw. | 180 |
| 44 | "Smart armband".tw. | 1 |
| 45 | bracelet*.tw. | 936 |
| 46 | Emotiv.tw. | 191 |
| 47 | NeuroSky.tw. | 149 |
| 48 | Mindo.tw. | 146 |
| 49 | StarLab.tw. | 6 |
| 50 | EmSense.tw. | 0 |
| 51 | "B-Alert X24".tw. | 0 |
| 52 | Enobio.tw. | 14 |
| 53 | BrainBit.tw. | 0 |
| 54 | NeuroSky.tw. | 18 |
| 55 | Muse.tw. | 1223 |
| 56 | OpenBCI.tw. | 21 |
| 57 | Neuroelectrics.tw. | 42 |
| 58 | "G.tec nautilus".tw. | 0 |
| 59 | BioSemi.tw. | 95 |
| 60 | "mBrainTrain".tw. | 1 |
| 61 | Cognionics.tw. | 8 |
| 62 | "CGX QUICK".tw. | 0 |
| 63 | Fitbit.tw. | 1473 |
| 64 | Garmin.tw. | 300 |
| 65 | "Misfit shine".tw. | 17 |
| 66 | "Polar loop".tw. | 28 |
| 67 | Jawbone.tw. | 825 |
| 68 | Geneactiv.tw. | 216 |
| 69 | Empatica.tw. | 83 |
| 70 | Amiigo.tw. | 3 |
| 71 | Actigraph.tw. | 4977 |
| 72 | "Apple Watch".tw. | 319 |
| 73 | Withings.tw. | 91 |
| 74 | Sensewear.tw. | 1075 |
| 75 | PowerWatch.tw. | 0 |
| 76 | "Samsung Galaxy Watch".tw. | 4 |
| 77 | Airofit.tw. | 1 |
| 78 | Amazfit.tw. | 6 |
| 79 | VivaLINK.tw. | 1 |
| 80 | "Wellue DuoEK".tw. | 0 |
| 81 | KardiaMobile.tw. | 52 |
| 82 | "Philips Biosensor".tw. | 2 |
| 83 | Biovitals.tw. | 3 |
| 84 | exp Mood Disorder/ | 225092 |
| 85 | mood disorder*.tw. | 299011 |
| 86 | exp Anxiety/ | 225063 |
| 87 | anxiety.tw. | 298928 |
| 88 | anxious.tw. | 23281 |
| 89 | 1 or 2 or 3 or 4 or 5 or 6 or 7 or 8 or 9 or 10 or 11 or 12 or 13 or 14 or 15 or 16 or 17 or 18 or 19 or 20 or 21 or 22 or 23 or 24 or 25 or 26 or 27 or 28 or 29 | 372011 |
| 90 | 30 or 31 or 32 or 33 or 34 or 35 or 36 or 37 or 38 or 39 or 40 or 41 or 42 or 43 or 44 or 45 or 46 or 47 or 48 or 49 or 50 or 51 or 52 or 53 or 54 or 55 or 56 or 57 or 58 or 59 or 60 or 61 or 62 or 63 or 64 or 65 or 66 or 67 or 68 or 69 or 70 or 71 or 72 or 73 or 74 or 75 or 76 or 77 or 78 or 79 or 80 or 81 or 82 or 83 | 59418 |
| 91 | 84 or 85 or 86 or 87 or 88 | 1527422 |
| 92 | 89 and 90 and 91 | 262 |
| 93 | limit 92 to (english language and yr="2015 -Current") | 247 |
| 94 | limit 93 to exclude medline journals | 32 |

Database(s): **APA PsycInfo**2002 to October Week 1 2022.

| **#** | **Searches** | **Results** |
| --- | --- | --- |
| 1 | exp Artificial Intelligence/ | 23781 |
| 2 | "Artificial Intelligence".tw. | 5106 |
| 3 | exp Machine Learning/ | 12327 |
| 4 | "Machine Learning".tw. | 9660 |
| 5 | exp Deep Learning/ | 0 |
| 6 | "Deep Learning".tw. | 2682 |
| 7 | "supervised learning".tw. | 1105 |
| 8 | "unsupervised learning".tw. | 728 |
| 9 | "reinforcement learning".tw. | 3117 |
| 10 | "Decision tree".tw. | 1395 |
| 11 | "K-Nearest Neighbor*".tw. | 577 |
| 12 | "Support vector machine*".tw. | 3176 |
| 13 | "Recurrent neural network*".tw. | 1081 |
| 14 | "convolutional neural network*".tw. | 1237 |
| 15 | "Artificial neural network*".tw. | 2025 |
| 16 | "Deep Neural Networks".tw. | 647 |
| 17 | "Naïve Bayes".tw. | 7 |
| 18 | "Naive Bayes".tw. | 605 |
| 19 | "Fuzzy Logic".tw. | 846 |
| 20 | "K-Means".tw. | 1536 |
| 21 | "Random Forest".tw. | 1111 |
| 22 | "Long Short-Term Memory Networks".tw. | 27 |
| 23 | autoencoder.tw. | 328 |
| 24 | "boltzmann machine".tw. | 134 |
| 25 | "deep belief network".tw. | 65 |
| 26 | "Gradient Boost*".tw. | 215 |
| 27 | AdaBoost.tw. | 341 |
| 28 | "Multilayer Perceptron".tw. | 118 |
| 29 | "Ensemble learning".tw. | 152 |
| 30 | exp Wearable Electronic Devices/ | 0 |
| 31 | wearable*.tw. | 2008 |
| 32 | "smart watch*".tw. | 45 |
| 33 | smartwatch*.tw. | 145 |
| 34 | acceleromet*.tw. | 4211 |
| 35 | gyroscop*.tw. | 108 |
| 36 | "inertial sensor".tw. | 53 |
| 37 | "inertial measurement unit*".tw. | 132 |
| 38 | "fitness band*".tw. | 2 |
| 39 | "flexible band*".tw. | 2 |
| 40 | headband*.tw. | 53 |
| 41 | "head band*".tw. | 3 |
| 42 | wristband*.tw. | 121 |
| 43 | "smart insole*".tw. | 1 |
| 44 | "Smart armband".tw. | 0 |
| 45 | bracelet*.tw. | 96 |
| 46 | Emotiv.tw. | 28 |
| 47 | NeuroSky.tw. | 13 |
| 48 | Mindo.tw. | 0 |
| 49 | StarLab.tw. | 1 |
| 50 | EmSense.tw. | 0 |
| 51 | "B-Alert X24".tw. | 0 |
| 52 | Enobio.tw. | 2 |
| 53 | BrainBit.tw. | 0 |
| 54 | NeuroSky.tw. | 13 |
| 55 | Muse.tw. | 301 |
| 56 | OpenBCI.tw. | 2 |
| 57 | Neuroelectrics.tw. | 1 |
| 58 | "G.tec nautilus".tw. | 0 |
| 59 | BioSemi.tw. | 15 |
| 60 | "mBrainTrain".tw. | 0 |
| 61 | Cognionics.tw. | 1 |
| 62 | "CGX QUICK".tw. | 0 |
| 63 | Fitbit.tw. | 245 |
| 64 | Garmin.tw. | 28 |
| 65 | "Misfit shine".tw. | 4 |
| 66 | "Polar loop".tw. | 1 |
| 67 | Jawbone.tw. | 23 |
| 68 | Geneactiv.tw. | 21 |
| 69 | Empatica.tw. | 56 |
| 70 | Amiigo.tw. | 0 |
| 71 | Actigraph.tw. | 1076 |
| 72 | "Apple Watch".tw. | 31 |
| 73 | Withings.tw. | 7 |
| 74 | Sensewear.tw. | 98 |
| 75 | PowerWatch.tw. | 0 |
| 76 | "Samsung Galaxy Watch".tw. | 0 |
| 77 | Airofit.tw. | 0 |
| 78 | Amazfit.tw. | 1 |
| 79 | VivaLINK.tw. | 0 |
| 80 | "Wellue DuoEK".tw. | 0 |
| 81 | KardiaMobile.tw. | 1 |
| 82 | "Philips Biosensor".tw. | 0 |
| 83 | Biovitals.tw. | 0 |
| 84 | exp Mood Disorder/ | 55645 |
| 85 | mood disorder*.tw. | 155082 |
| 86 | exp Anxiety/ | 225063 |
| 87 | anxiety.tw. | 298928 |
| 88 | anxious.tw. | 23281 |
| 89 | 1 or 2 or 3 or 4 or 5 or 6 or 7 or 8 or 9 or 10 or 11 or 12 or 13 or 14 or 15 or 16 or 17 or 18 or 19 or 20 or 21 or 22 or 23 or 24 or 25 or 26 or 27 or 28 or 29 | 36989 |
| 90 | 30 or 31 or 32 or 33 or 34 or 35 or 36 or 37 or 38 or 39 or 40 or 41 or 42 or 43 or 44 or 45 or 46 or 47 or 48 or 49 or 50 or 51 or 52 or 53 or 54 or 55 or 56 or 57 or 58 or 59 or 60 or 61 or 62 or 63 or 64 or 65 or 66 or 67 or 68 or 69 or 70 or 71 or 72 or 73 or 74 or 75 or 76 or 77 or 78 or 79 or 80 or 81 or 82 or 83 | 7423 |
| 91 | 84 or 85 or 86 or 87 or 88 | 485921 |
| 92 | 89 and 90 and 91 | 27 |
| 93 | limit 92 to (english language and yr="2015 -Current") | 19 |

**CINHAL**: Monday, October 3, 2022 4:10:26 PM

| **#** | **Query** | **Results** |
| --- | --- | --- |
| S88 | Narrow by Language: - english | 14 |
| S87 | Limiters - Date Published: 20150101-20221231 | 14 |
| S86 | S82 AND S83 AND S84 | 14 |
| S85 | (S78 OR S79 OR S80 OR S81 OR S82) | 354,718 |
| S84 | (S30 OR S31 OR S32 OR S33 OR S34 OR S35 OR S36 OR S37 OR S38 OR S39 OR S40 OR S41 OR S42 OR S43 OR S44 OR S45 OR S46 OR S47 OR S48 OR S49 OR S50 OR S51 OR S52 OR S53 OR S54 OR S55 OR S56 OR S57 OR S58 OR S59 OR S60 OR S61 OR S62 OR S63 OR S64 OR S65 OR S66 OR S67 OR S68 OR S69 OR S70 OR S71 OR S72 OR S73 OR S74 OR S75 OR S76 OR S77) | 13,841 |
| S83 | (S1 OR S2 OR S3 OR S4 OR S5 OR S6 OR S7 OR S8 OR S9 OR S10 OR S11 OR S12 OR S13 OR S14 OR S15 OR S16 OR S17 OR S18 OR S19 OR S20 OR S21 OR S22 OR S23 OR S24 OR S25 OR S26 OR S27 OR S28 OR S29) | 25,112 |
| S82 | AB anxious | 6,381 |
| S81 | AB anxiety | 86,967 |
| S80 | MW anxiety | 69,710 |
| S79 | AB Mood Disorder* | 87,122 |
| S78 | MW Mood Disorder | 69,864 |
| S77 | AB Biovitals | 2 |
| S76 | AB "Philips Biosensor" | 0 |
| S75 | AB KardiaMobile | 13 |
| S74 | AB "Wellue DuoEK" | 0 |
| S73 | AB VivaLINK | 0 |
| S72 | AB Amazfit | 1 |
| S71 | AB Airofit | 0 |
| S70 | AB "Samsung Galaxy Watch" | 2 |
| S69 | AB PowerWatch | 1 |
| S68 | AB Sensewear | 237 |
| S67 | AB Withings | 43 |
| S66 | AB "Apple Watch" | 115 |
| S65 | AB Actigraph | 1,779 |
| S64 | AB Amiigo | 0 |
| S63 | AB Empatica | 74 |
| S62 | AB Geneactiv | 87 |
| S61 | AB Jawbone | 230 |
| S60 | AB "Polar loop" | 5 |
| S59 | AB "Misfit shine" | 9 |
| S58 | AB Garmin | 92 |
| S57 | AB Garmin | 0 |
| S56 | AB Fitbit | 459 |
| S55 | AB "CGX QUICK" | 0 |
| S54 | AB Cognionics | 0 |
| S53 | AB "mBrainTrain" | 0 |
| S52 | AB BioSemi | 4 |
| S51 | AB "G.tec nautilus" | 0 |
| S50 | AB Neuroelectrics | 46 |
| S49 | AB Mindo | 0 |
| S48 | AB NeuroSky | 3 |
| S47 | AB Emotiv | 12 |
| S46 | AB bracelet* | 254 |
| S45 | AB "armband*" | 280 |
| S44 | AB "Smart armband*" | 0 |
| S43 | AB "smart headband*" | 0 |
| S42 | AB "smart insole*" | 1 |
| S41 | AB wristband* | 248 |
| S40 | AB "head band*" | 21 |
| S39 | AB headband* | 102 |
| S38 | AB "fitness band*" | 12 |
| S37 | AB "inertial measurement unit*" | 418 |
| S36 | AB "inertial sensor" | 196 |
| S35 | AB gyroscop | 0 |
| S34 | AB acceleromet* | 7,796 |
| S33 | AB smartwatch* | 189 |
| S32 | AB "smart watch*" | 59 |
| S31 | AB wearable* | 3,069 |
| S30 | MW wearable devices | 91 |
| S29 | AB "Ensemble learning" | 103 |
| S28 | AB "Multilayer Perceptron" | 195 |
| S27 | AB AdaBoost | 131 |
| S26 | AB "Gradient Boost*" | 564 |
| S25 | AB "deep belief network" | 24 |
| S24 | AB "boltzmann machine" | 10 |
| S23 | AB autoencoder | 93 |
| S22 | AB "Long Short-Term Memory Networks" | 15 |
| S21 | AB "Random Forest" | 2,006 |
| S20 | AB "K-Means" | 972 |
| S19 | AB "Fuzzy Logic" | 207 |
| S18 | AB "Naive Bayes" | 175 |
| S17 | AB "Naïve Bayes" | 256 |
| S16 | AB "Deep Neural Networks" | 147 |
| S15 | AB "Artificial neural network*" | 1,195 |
| S14 | AB "convolutional neural network*" | 1,359 |
| S13 | AB "Recurrent neural network*" | 182 |
| S12 | AB "Support vector machine*" | 2,429 |
| S11 | AB "K-Nearest Neighbor*" | 412 |
| S10 | AB "Decision tree" | 2,352 |
| S9 | AB "reinforcement learning" | 272 |
| S8 | AB "unsupervised learning" | 107 |
| S7 | AB "supervised learning" | 250 |
| S6 | AB "deep Learning" | 2,688 |
| S5 | MW "deep Learning" | 999 |
| S4 | AB "Machine Learning" | 7,913 |
| S3 | MW "Machine Learning" | 3,155 |
| S2 | AB "Artificial Intelligence" | 4,314 |
| S1 | MW Artificial Intelligence | 7,059 |

| **Database** | **Query** | **Results** |
| --- | --- | --- |
| **Scopus** | ( TITLE-ABS-KEY ( "Artificial Intelligence" OR "Machine Learning" OR "Deep Learning" OR "supervised learning" OR "unsupervised learning" OR "reinforcement learning" OR "Decision tree" OR "K-Nearest Neighbor*" OR "Support vector machine*" OR "Recurrent neural network*" OR "convolutional neural network*" OR "Artificial neural network*" OR "Deep Neural Networks" OR "Naïve Bayes" OR "Naive Bayes" OR "Fuzzy Logic" OR "K-Means" OR "Random Forest" OR "Long Short-Term Memory Networks" OR autoencoder OR "boltzmann machine" OR "deep belief network" OR "Gradient Boost*" OR adaboost OR "Multilayer Perceptron" OR "Ensemble learning" ) ) AND ( TITLE-ABS-KEY ( wearable* OR "smart watch*" OR smartwatch* OR acceleromet* OR gyroscop* OR "inertial sensor" OR "inertial measurement unit*" OR "fitness band*" OR "flexible band*" OR headband* OR "head band*" OR wristband* OR "smart insole*" OR "Smart armband" OR bracelet* OR emotiv OR neurosky OR mindo OR starlab OR emsense OR "B-Alert X24" OR enobio OR brainbit OR muse OR openbci OR neuroelectrics OR "G.tec nautilus" OR biosemi OR "mBrainTrain" OR cognionics OR "CGX QUICK" OR fitbit OR garmin OR "Misfit shine" OR "Polar loop" OR jawbone OR geneactiv OR empatica OR amiigo OR actigraph OR "Apple Watch" OR withings OR sensewear OR powerwatch OR "Samsung Galaxy Watch" OR airofit OR amazfit OR vivalink OR "Wellue DuoEK" OR kardiamobile OR "Philips Biosensor" OR biovitals ) ) AND ( TITLE-ABS-KEY (mood disorder* OR anxiety OR anxious) ) AND ( LIMIT-TO ( LANGUAGE , "English" ) ) AND ( LIMIT-TO ( PUBYEAR , 2022 ) OR LIMIT-TO ( PUBYEAR , 2021 ) OR LIMIT-TO ( PUBYEAR , 2020 ) OR LIMIT-TO ( PUBYEAR , 2019 ) OR LIMIT-TO ( PUBYEAR , 2018 ) OR LIMIT-TO ( PUBYEAR , 2017 ) OR LIMIT-TO ( PUBYEAR , 2016 ) OR LIMIT-TO ( PUBYEAR , 2015 ) ) AND ( LIMIT-TO ( DOCTYPE , "ar" ) OR LIMIT-TO ( DOCTYPE , "cp" ) OR LIMIT-TO ( DOCTYPE , "ch" ) ) | 584 |
| **IEEE Xplore** | ("Abstract":"Artificial Intelligence" OR "Abstract":"Machine Learning" OR "Abstract":"Deep Learning" OR "Abstract":"supervised learning" OR "Abstract":"unsupervised learning" OR "Abstract":"reinforcement learning" OR "Abstract":"Decision tree" OR "Abstract":"K-Nearest Neighbor" OR "Abstract":"Support vector machine" OR "Abstract":"Recurrent neural network" OR "Abstract":"convolutional neural network" OR "Abstract":"Artificial neural network" OR "Abstract":"Deep Neural Network" OR "Abstract":"Naïve Bayes" OR "Abstract":"Naive Bayes" OR "Abstract":"Fuzzy Logic" OR "Abstract":"K-Means" OR "Abstract":"Random Forest" OR "Abstract":"Long Short-Term Memory Networks" OR "Abstract":autoencoder OR "Abstract":"boltzmann machine" OR "Abstract":"deep belief network" OR "Abstract":"Gradient Boost" OR "Abstract":AdaBoost OR "Abstract":"Multilayer Perceptron" OR "Abstract":"Ensemble learning") AND ("Abstract":wearable OR "Abstract":wearable OR "Abstract":"smart watch" OR "Abstract":smartwatch OR "Abstract":"smart watches" OR "Abstract":smartwatches OR "Abstract":acceleromet OR "Abstract":gyroscop OR "Abstract":"inertial sensor" OR "Abstract":"inertial measurement unit" OR "Abstract":"fitness band" OR "Abstract":"flexible band" OR "Abstract":headband OR "Abstract":"head band" OR "Abstract":wristband OR "Abstract":"smart insole" OR "Abstract":"Smart armband" OR "Abstract":bracelet* OR "Abstract":Emotiv OR "Abstract":NeuroSky OR "Abstract":Mindo StarLab OR "Abstract":EmSense OR "Abstract":"B-Alert X24" OR "Abstract":Enobio OR "Abstract":BrainBit OR "Abstract":Muse OR "Abstract":OpenBCI OR "Abstract":Neuroelectrics OR "Abstract":"G.tec nautilus" OR "Abstract":BioSemi OR "Abstract":"mBrainTrain" OR "Abstract":Cognionics OR "Abstract":"CGX QUICK" OR "Abstract":Fitbit OR "Abstract":Garmin OR "Abstract":"Misfit shine" OR "Abstract":"Polar loop" OR "Abstract":Jawbone OR "Abstract":Geneactiv OR "Abstract":Empatica OR "Abstract":Amiigo OR "Abstract":Actigraph OR "Abstract":"Apple Watch" OR "Abstract":Withings OR "Abstract":Sensewear OR "Abstract":PowerWatch OR "Abstract":"Samsung Galaxy Watch" OR "Abstract":Airofit OR "Abstract":Amazfit OR "Abstract":VivaLINK OR "Abstract":"Wellue DuoEK" OR "Abstract":KardiaMobile OR "Abstract":"Philips Biosensor" OR "Abstract":Biovitals) AND ("Abstract": mood disorder* OR "Abstract": anxiety OR "Abstract": anxious) | 20 |
| **ACM Digital library** | [[Abstract: "artificial intelligence"] OR [Abstract: "machine learning"] OR [Abstract: "deep learning"] OR [Abstract: "supervised learning"] OR [Abstract: "unsupervised learning"] OR [Abstract: "reinforcement learning"] OR [Abstract: "decision tree"] OR [Abstract: "k-nearest neighbor*"] OR [Abstract: "support vector machine*"] OR [Abstract: "recurrent neural network*"] OR [Abstract: "convolutional neural network*"] OR [Abstract: "artificial neural network*"] OR [Abstract: "deep neural networks"] OR [Abstract: "naïve bayes"] OR [Abstract: "naive bayes"] OR [Abstract: "fuzzy logic"] OR [Abstract: "k-means"] OR [Abstract: "random forest"] OR [Abstract: "long short-term memory networks"] OR [Abstract: autoencoder] OR [Abstract: "boltzmann machine"] OR [Abstract: "deep belief network"] OR [Abstract: "gradient boost*"] OR [Abstract: adaboost] OR [Abstract: "multilayer perceptron"] OR [Abstract: "ensemble learning"]] AND [[Abstract: wearable*] OR [Abstract: "smart watch*"] OR [Abstract: smartwatch*] OR [Abstract: acceleromet*] OR [Abstract: gyroscop*] OR [Abstract: "inertial sensor"] OR [Abstract: "inertial measurement unit*"] OR [Abstract: "fitness band*"] OR [Abstract: "flexible band*"] OR [Abstract: headband*] OR [Abstract: "head band*"] OR [Abstract: wristband*] OR [Abstract: "smart insole*"] OR [Abstract: "smart armband"] OR [Abstract: bracelet*] OR [Abstract: emotiv] OR [Abstract: neurosky] OR [Abstract: mindo starlab] OR [Abstract: emsense] OR [Abstract: "b-alert x24"] OR [Abstract: enobio] OR [Abstract: brainbit] OR [Abstract: muse] OR [Abstract: openbci] OR [Abstract: neuroelectrics] OR [Abstract: "g.tec nautilus"] OR [Abstract: biosemi] OR [Abstract: "mbraintrain"] OR [Abstract: cognionics] OR [Abstract: "cgx quick"] OR [Abstract: fitbit] OR [Abstract: garmin] OR [Abstract: "misfit shine"] OR [Abstract: "polar loop"] OR [Abstract: jawbone] OR [Abstract: geneactiv] OR [Abstract: empatica] OR [Abstract: amiigo] OR [Abstract: actigraph] OR [Abstract: "apple watch"] OR [Abstract: withings] OR [Abstract: sensewear] OR [Abstract: powerwatch] OR [Abstract: "samsung galaxy watch"] OR [Abstract: airofit] OR [Abstract: amazfit] OR [Abstract: vivalink] OR [Abstract: "wellue duoek"] OR [Abstract: kardiamobile] OR [Abstract: "philips biosensor"] OR [Abstract: biovitals]] AND [[Abstract: mood disorder*] OR [Abstract: anxiety*] OR [Abstract: anxious *]] AND [Publication Date: (01/01/2015 TO 12/31/2022)] | 14 |
| **Google Scholar** | ("Artificial Intelligence" OR "Machine Learning" OR "Deep Learning") AND (wearable* OR smartwatch* OR Emotiv OR Mindo OR Muse OR Fitbit OR Garmin OR Geneactiv OR Empatica "Apple Watch" OR "Polar loop") AND (mood disorder* OR anxiety OR anxious) | 100 |
