# Supplementary material for: Wearable Artificial Intelligence for Detecting Anxiety: Systematic Review and Meta-Analysis
Source: J Med Internet Res. 2023 Nov 8;25:e48754. doi: 10.2196/48754 (PMC10666012; doi:10.2196/48754)
Supplement: Multimedia Appendix 3 [file jmir_v25i1e48754_app3.docx]

| **Extracted data** | **Definition** |
| --- | --- |
| **Study Characteristics** |  |
| Author | The first author of the study. |
| Year of publication | The year in which the study was published. |
| Country of publication | The country where the study was published. |
| Type of publication | The venue where the study was published: peer-reviewed journal articles, book chapters, dissertations, or conference proceedings |
| Number of participants | What is the number of participants from which the data was collected? |
| Mean age (range) | What is the mean/range age of the participants? |
| Female percentage | What is the female percentage of the participants? |
| Participants Health Conditions | What is the health condition of the participants? |
| **Wearable AI characteristics** |  |
| Name of the wearable device | What is the name of the wearable device (e.g., Fitbit, Empatica, ApplyWatch, ActiWatch, etc..)? |
| Placement of the wearable device | Where the wearable device is worn during the experiment in paper or normally (wrist, chest, head, ears, forehead, eyes, fingers, foot, etc..)? |
| Aim of AI algorithm | What was the algorithm used for (diagnosis, screening, monitoring, treatment, prevention, etc.))? |
| Problem solving approaches | What is the problem-solving approach that the algorithm follows (Classification, regression)? |
| AI algorithm used | What are the main AI algorithms/models (e.g., RF, SVM, ANN, CNN, RNN, DNN, k-NN, MLP, DBN, DBM, DPN BN, CRT, DT, LASSO, LR, MFA, MLR, MDL, NB, NN, NSC, RBFN) used in the paper? |
| Data sources | What is the source of data that was used for developing the algorithms (open source or closed source)? |
| Data input | What is the data that was used for developing the algorithm? |
| Ground truth assessment | How the actual status (e.g., diagnosis) of the user was confirmed (questionnaire (PHQ-9), interview, test, etc..)? |
| Type of validation | What is the approach that was used to validate the developed algorithm (e.g., Training-test split, K-fold cross-validation, Nested Cross-Validation, Leave One Out cross-validation, Apparent validation, external validation)? |
| Performance measures used | What are the measures used to assess the performance of the algorithm (accuracy, sensitivity (recall), specificity, precision, AUC, etc...)? |
| Results | The highest results for each performance measure for each algorithm. Calculate the measures if the confusion matrix is reported. |
