# Supplementary material for: Wearable Artificial Intelligence for Detecting Anxiety: Systematic Review and Meta-Analysis
Source: J Med Internet Res. 2023 Nov 8;25:e48754. doi: 10.2196/48754 (PMC10666012; doi:10.2196/48754)
Supplement: Multimedia Appendix 5 [file jmir_v25i1e48754_app5.docx]

Multimedia Appendix 5: Characteristics of each included study

| Study ^Ref^ | Year | Publication type | Country | Sample size | Mean  age | Age  range | Female % | Participants’ health condition |
| --- | --- | --- | --- | --- | --- | --- | --- | --- |
| Arsalan ^35^ | 2020 | Conference Paper | Pakistan | 28 | NR | 18-40 | 53.6 | General |
| Arsalan ^36^ | 2021 | Journal article | Pakistan | 65 | 27.5 | NR | 49 | General |
| Coutts ^37^ | 2020 | Journal article | United Kingdom | 668 | 21.9 | 18-69 | 71.2 | General |
| Feng ^38^ | 2020 | Conference Paper | United States | 97 | NR | NR | 74 | General |
| Fukuda ^39^ | 2020 | Conference Paper | Japan | 60 | NR | NR | NR | General |
| Gu ^40^ | 2017 | Journal article | China | 22 | NR | NR | NR | General |
| Ihmig ^41^ | 2020 | Journal article | Germany | 57 | NR | 18-40 | NR | Arachnophobia |
| Jacobson ^42^ | 2021 | Journal article | United States | 265 | 44.3 | 25-72 | 58.1 | General |
| Jacobson ^43^ | 2022 | Journal article | United States | 264 | 29.8 | 20-39 | 48.5 | General |
| Jin ^44^ | 2020 | Journal article | China | 60 | NR | 18-26 | 50 | General |
| Khan ^45^ | 2021 | Journal article | Pakistan | 10 | NR | 20-50 | 90 | General |
| Miranda ^46^ | 2016 | Journal article | Mexico | 10 | 24.7 | 23-26 | 50 | General |
| Nath ^47^ | 2021 | Journal article | United States | 41 | 73.4 | 60-80 | 63.4 | General |
| Nishimura ^48^ | 2022 | Conference Paper | Japan | 100 | 42.1 | NR | 37 | General |
| Rother ^49^ | 2019 | Conference Paper | United Kingdom | 823 | NR | NR | NR | General |
| Saha ^50^ | 2021 | Journal article | United States | 754 | 34.9 | 20-68 | NR | General |
| Šalkevicius ^51^ | 2019 | Journal article | Lithuania | 30 | 27.5 | 21-34 | 43.3 | Glossophobia, healthy |
| Shaukat-Jali ^52^ | 2021 | Journal article | United Kingdom | 12 | 19.8 | NR | 58 | Social anxiety |
| Tiwari ^53^ | 2019 | Conference Paper | United States | 196 | 38.6 | NR | 66.3 | General |
| Tsai ^54^ | 2022 | Journal article | Taiwan | 59 | NR | 20-74 | 61 | Panic disorders |
| Zheng ^55^ | 2016 | Journal article | Hong Kong | 20 | NR | 18-23 | NR | General |
| NR: not reported | | | | | | | | |

1. Arsalan, A., Majid, M. & Anwar, S. M. in *Intelligent Technologies and Applications.* (eds Imran Sarwar Bajwa, Tatjana Sibalija, & Dayang Norhayati Abang Jawawi) 187-197 (Springer Singapore).
2. Arsalan, A. & Majid, M. A study on multi-class anxiety detection using wearable EEG headband. *Journal of Ambient Intelligence and Humanized Computing*, doi:10.1007/s12652-021-03249-y (2021).
3. Coutts, L. V., Plans, D., Brown, A. W. & Collomosse, J. Deep learning with wearable based heart rate variability for prediction of mental and general health. *J Biomed Inform* **112**, 103610, doi:10.1016/j.jbi.2020.103610 (2020).
4. Feng, T. & Narayanan, S. S. in *ICASSP 2020 - 2020 IEEE International Conference on Acoustics, Speech and Signal Processing (ICASSP).* 1011-1015.
5. Fukuda, S. *et al.* in *2020 IEEE International Conference on Pervasive Computing and Communications Workshops (PerCom Workshops).* 1-6.
6. Gu, J. *et al.* Wearable Social Sensing: Content-Based Processing Methodology and Implementation. *IEEE Sensors Journal* **17**, 7167-7176, doi:10.1109/JSEN.2017.2754289 (2017).
7. Ihmig, F. R. *et al.* On-line anxiety level detection from biosignals: Machine learning based on a randomized controlled trial with spider-fearful individuals. *PLoS One* **15**, e0231517, doi:10.1371/journal.pone.0231517 (2020).
8. Jacobson, N. C., Lekkas, D., Huang, R. & Thomas, N. Deep learning paired with wearable passive sensing data predicts deterioration in anxiety disorder symptoms across 17-18 years. *J Affect Disord* **282**, 104-111, doi:10.1016/j.jad.2020.12.086 (2021).
9. Jacobson, N. C. & Feng, B. Digital phenotyping of generalized anxiety disorder: using artificial intelligence to accurately predict symptom severity using wearable sensors in daily life. *Transl Psychiatry* **12**, 336, doi:10.1038/s41398-022-02038-1 (2022).
10. Jin, J. *et al.* Attention-Block Deep Learning Based Features Fusion in Wearable Social Sensor for Mental Wellbeing Evaluations. *IEEE Access* **8**, 89258-89268, doi:10.1109/ACCESS.2020.2994124 (2020).
11. Khan, N. S., Ghani, M. S. & Anjum, G. ADAM-sense: Anxiety-displaying activities recognition by motion sensors. *Pervasive and Mobile Computing* **78**, 101485, doi:https://doi.org/10.1016/j.pmcj.2021.101485 (2021).
12. Miranda, D., Favela, J., Ibarra, C. & Cruz, N. Naturalistic Enactment to Elicit and Recognize Caregiver State Anxiety. *J Med Syst* **40**, 192, doi:10.1007/s10916-016-0551-0 (2016).
13. Nath, R. K. & Thapliyal, H. Machine Learning-Based Anxiety Detection in Older Adults Using Wristband Sensors and Context Feature. *SN Computer Science* **2**, 359, doi:10.1007/s42979-021-00744-z (2021).
14. Nishimura, Y. *et al.* in *Sensor-and Video-Based Activity and Behavior Computing* 1-26 (Springer, 2022).
15. Rother, R., Sun, Y. & Lo, B. in *Living in the Internet of Things (IoT 2019).* 1-6.
16. Saha, K. *et al.* Person-Centered Predictions of Psychological Constructs with Social Media Contextualized by Multimodal Sensing. *Proc. ACM Interact. Mob. Wearable Ubiquitous Technol.* **5**, Article 32, doi:10.1145/3448117 (2021).
17. Šalkevicius, J., Damaševičius, R., Maskeliunas, R. & Laukienė, I. Anxiety Level Recognition for Virtual Reality Therapy System Using Physiological Signals. *Electronics* **8**, doi:10.3390/electronics8091039 (2019).
18. Shaukat-Jali, R., van Zalk, N. & Boyle, D. E. Detecting Subclinical Social Anxiety Using Physiological Data From a Wrist-Worn Wearable: Small-Scale Feasibility Study. *JMIR Form Res* **5**, e32656, doi:10.2196/32656 (2021).
19. Tiwari, A., Cassani, R., Narayanan, S. & Falk, T. H. in *2019 41st Annual International Conference of the IEEE Engineering in Medicine and Biology Society (EMBC).* 2213-2216.
20. Tsai, C. H. *et al.* Panic Attack Prediction Using Wearable Devices and Machine Learning: Development and Cohort Study. *JMIR Med Inform* **10**, e33063, doi:10.2196/33063 (2022).
21. Zheng, Y., Wong, T. C. H., Leung, B. H. K. & Poon, C. C. Y. Unobtrusive and Multimodal Wearable Sensing to Quantify Anxiety. *IEEE Sensors Journal* **16**, 3689-3696, doi:10.1109/JSEN.2016.2539383 (2016).
