# Supplementary material for: Wearable Artificial Intelligence for Detecting Anxiety: Systematic Review and Meta-Analysis
Source: J Med Internet Res. 2023 Nov 8;25:e48754. doi: 10.2196/48754 (PMC10666012; doi:10.2196/48754)
Supplement: Multimedia Appendix 6 [file jmir_v25i1e48754_app6.docx]

**Appendix 6: Features of wearable AI**

| Study ^Ref^ | Name of WD | Placement of WD | Aim of AI algorithm | Problem solving approach | AI algorithm | Dataset source | Data input | Ground truth assessment | Validation approach |
| --- | --- | --- | --- | --- | --- | --- | --- | --- | --- |
| Arsalan ^35^ | MUSE | Head | Detection | Classification, Regression | LogR, MLP, RF | Closed | EEG data | STAI | K-fold |
| Arsalan ^36^ | MUSE | Head | Detection | Classification | LogR, MLP, RF | Closed | EEG data | STAI | K-fold |
| Coutts ^37^ | Biobeam | Wrist | Detection | Classification | LSTM | Closed | Heart rate data | DASS, STAI | Hold-out |
| Feng ^38^ | Fitbit Charge 2 | Wrist | Detection | Classification, clustering | SVM | Open | Activity data,  heart rate data | STAI | K-fold |
| Fukuda ^39^ | Fitbit Charge 3 | Wrist | Detection | Classification | RF | Closed | Sleep data | DAMS | LOOCV |
| Gu ^40^ | NR | Neck, wrist | Detection | Classification | K-means | Closed | Audio data | STAI | NR |
| Ihmig ^41^ | BITalino, Rythem+ | Arm, hand | Detection | Classification | DT, Ensemble model, KNN, LDA, NB, QDA, SVM | Closed | EDA data,  heart rate data | Observation, self-rated question | K-fold |
| Jacobson ^42^ | Mini Mitter Actiwatch | Wrist | Detection | Classification | ANN, GLM, GP, KNN, RF, RR, SR, SVM, XGBoost | Closed | Activity data,  sleep data | CIDI | K-fold |
| Jacobson ^43^ | ActiGraph | Hip | Detection | Classification, regression | Ensemble model | Closed | Activity data | CIDI | K-fold |
| Jin ^44^ | NR | Wrist | Detection | Classification | CNN, LSTM | Closed | Activity data,  audio data | STAI | Hold-out |
| Khan ^45^ | NR | Wrist | Detection | Classification | CNN, DNN, GB, LSTM, RF | Closed | Activity data | Clinician assessment | Hold-out |
| Miranda ^46^ | Empatica E3, Muse, Zephyr HxM | Chest, head, wrist | Detection | Classification | SVM | Closed | EDA data,  heart rate data | Observation | Hold-out, LOOCV |
| Nath ^47^ | NR | Wrist | Detection | Classification | LogR, RF, SVM | Closed | EDA data,  heart rate data | STAI | Hold-out |
| Nishimura ^48^ | Fitbit Charge 3 | Wrist | Detection | Classification | LightGBM | Closed | Activity data, behavioural data, heart rate data, sleep data, weather data | DAMS | K-fold |
| Rother ^49^ | NR | Waist | Detection | Classification | DT, MLP, SVM | Closed | Activity data,  EDA data, heart rate data, respiratory rate measures, social interaction | NR | NR |
| Saha ^50^ | Vivosmart | Wrist | Detection | Clustering, regression | elasticNet, HC, GBR, K-means, MLP, PAM, RR, SVR, XGBoost | Open | Activity data, behavioural data, location, sleep data, smartphone usage data, social media data | STAI | Hold-out, K-fold |
| Šalkevicius ^51^ | Empatica E4 | Wrist | Detection | Classification | SVM | Closed | EDA data, heart rate data, skin temperature | SUDS | K-fold, LOOCV |
| Shaukat-Jali ^52^ | Empatica E4 | Wrist | Detection | Classification | DT, KNN, RF, SVM | Closed | EDA data, heart rate data, skin temperature | LSAS, SPSQ | K-fold |
| Tiwari ^53^ | Fitbit Charge 2, Omsignal Smart-Shirt | Chest, wrist | Detection | Classification | SVM | Closed | Heart rate data | NR | K-fold |
| Tsai ^54^ | Vivosmart 4 | Wrist | Prediction | Classification | Adaboost, DT, LDA, RF, RGF, XGBoost | Closed | Activity data, anxiety level, depression level, environmental data, heart rate data, sleep data | PDSS | K-fold |
| Zheng ^55^ | MindWave Mobile | Eyes, head | Detection | Classification | KNN, SVM | Closed | EEG data, heart rate data | CSAI-2 | Hold-out |
| ANN: Artificial Neural Network; BDI-II: Beck Depression Inventory-II; BPRS: Brief Psychiatric Rating Scale; BT: Boosted Trees; CIDI: Composite international diagnostic interview; CNN: Convolutional Neural Network; DAMS: Depression and Anxiety Mood Scale; DASS: Depression Anxiety Stress Scales; DNN: Deep Neural Network; DSM: Diagnostic and Statistical Manual of Mental Health; DT: Decision tree; EDA: Electrodermal activity ; EEG: Electroencephalograph; GMM: Gaussian mixture models; KNN: K-Nearest Neighbors; LDA: Linear discriminant analysis; LightGBM: Light Gradient Boosting Machine; LinR: Linear regression; LogR: Logistic regression; LOOCV: Leave-One-Out Cross-Validation; LSAS: Liebowitz Social Anxiety Scale; NB: Naive Bayes; NR: Not reported; PDSS: Panic Disorder Severity Scale; QDA: Quadratic Discriminant Analysis ; RF: Random Forest; RR: Ridge Regression; SPSQ: Social Phobia Screening Questionnaire; STAI: State-Trait Anxiety Inventory; SUDS: Subjective units of distress scale; SVM: Support Vector Machine; SVR: Support Vector regressor; XGBoost: extreme gradient boosting | | | | | | | | | |

1. Arsalan, A., Majid, M. & Anwar, S. M. in *Intelligent Technologies and Applications.* (eds Imran Sarwar Bajwa, Tatjana Sibalija, & Dayang Norhayati Abang Jawawi) 187-197 (Springer Singapore).
2. Arsalan, A. & Majid, M. A study on multi-class anxiety detection using wearable EEG headband. *Journal of Ambient Intelligence and Humanized Computing*, doi:10.1007/s12652-021-03249-y (2021).
3. Coutts, L. V., Plans, D., Brown, A. W. & Collomosse, J. Deep learning with wearable based heart rate variability for prediction of mental and general health. *J Biomed Inform* **112**, 103610, doi:10.1016/j.jbi.2020.103610 (2020).
4. Feng, T. & Narayanan, S. S. in *ICASSP 2020 - 2020 IEEE International Conference on Acoustics, Speech and Signal Processing (ICASSP).* 1011-1015.
5. Fukuda, S., Matsuda, Y., Tani, Y., Arakawa, Y. & Yasumoto, K. in *2020 IEEE International Conference on Pervasive Computing and Communications Workshops (PerCom Workshops).* 1-6.
6. Gu, J. *et al.* Wearable Social Sensing: Content-Based Processing Methodology and Implementation. *IEEE Sensors Journal* **17**, 7167-7176, doi:10.1109/JSEN.2017.2754289 (2017).
7. Ihmig, F. R. *et al.* On-line anxiety level detection from biosignals: Machine learning based on a randomized controlled trial with spider-fearful individuals. *PLoS One* **15**, e0231517, doi:10.1371/journal.pone.0231517 (2020).
8. Jacobson, N. C., Lekkas, D., Huang, R. & Thomas, N. Deep learning paired with wearable passive sensing data predicts deterioration in anxiety disorder symptoms across 17-18 years. *J Affect Disord* **282**, 104-111, doi:10.1016/j.jad.2020.12.086 (2021).
9. Jacobson, N. C. & Feng, B. Digital phenotyping of generalized anxiety disorder: using artificial intelligence to accurately predict symptom severity using wearable sensors in daily life. *Transl Psychiatry* **12**, 336, doi:10.1038/s41398-022-02038-1 (2022).
10. Jin, J. *et al.* Attention-Block Deep Learning Based Features Fusion in Wearable Social Sensor for Mental Wellbeing Evaluations. *IEEE Access* **8**, 89258-89268, doi:10.1109/ACCESS.2020.2994124 (2020).
11. Khan, N. S., Ghani, M. S. & Anjum, G. ADAM-sense: Anxiety-displaying activities recognition by motion sensors. *Pervasive and Mobile Computing* **78**, 101485, doi:https://doi.org/10.1016/j.pmcj.2021.101485 (2021).
12. Miranda, D., Favela, J., Ibarra, C. & Cruz, N. Naturalistic Enactment to Elicit and Recognize Caregiver State Anxiety. *J Med Syst* **40**, 192, doi:10.1007/s10916-016-0551-0 (2016).
13. Nath, R. K. & Thapliyal, H. Machine Learning-Based Anxiety Detection in Older Adults Using Wristband Sensors and Context Feature. *SN Computer Science* **2**, 359, doi:10.1007/s42979-021-00744-z (2021).
14. 14 Nishimura, Y. *et al.* in *Sensor-and Video-Based Activity and Behavior Computing* 1-26 (Springer, 2022).
15. Rother, R., Sun, Y. & Lo, B. in *Living in the Internet of Things (IoT 2019).* 1-6.
16. Saha, K. *et al.* Person-Centered Predictions of Psychological Constructs with Social Media Contextualized by Multimodal Sensing. *Proc. ACM Interact. Mob. Wearable Ubiquitous Technol.* **5**, Article 32, doi:10.1145/3448117 (2021).
17. Šalkevicius, J., Damaševičius, R., Maskeliunas, R. & Laukienė, I. Anxiety Level Recognition for Virtual Reality Therapy System Using Physiological Signals. *Electronics* **8**, doi:10.3390/electronics8091039 (2019).
18. Shaukat-Jali, R., van Zalk, N. & Boyle, D. E. Detecting Subclinical Social Anxiety Using Physiological Data From a Wrist-Worn Wearable: Small-Scale Feasibility Study. *JMIR Form Res* **5**, e32656, doi:10.2196/32656 (2021).
19. Tiwari, A., Cassani, R., Narayanan, S. & Falk, T. H. in *2019 41st Annual International Conference of the IEEE Engineering in Medicine and Biology Society (EMBC).* 2213-2216.
20. Tsai, C. H. *et al.* Panic Attack Prediction Using Wearable Devices and Machine Learning: Development and Cohort Study. *JMIR Med Inform* **10**, e33063, doi:10.2196/33063 (2022).
21. Zheng, Y., Wong, T. C. H., Leung, B. H. K. & Poon, C. C. Y. Unobtrusive and Multimodal Wearable Sensing to Quantify Anxiety. *IEEE Sensors Journal* **16**, 3689-3696, doi:10.1109/JSEN.2016.2539383 (2016).
