# Supplementary material for: Wearable Artificial Intelligence for Detecting Anxiety: Systematic Review and Meta-Analysis
Source: J Med Internet Res. 2023 Nov 8;25:e48754. doi: 10.2196/48754 (PMC10666012; doi:10.2196/48754)
Supplement: Multimedia Appendix 7 [file jmir_v25i1e48754_app7.docx]

**Multimedia Appendix 7: Reviewers’ judgments about each domain in “risk of bias” and "applicability concerns" for each included study**
